# Nutrient composition and safety evaluation of simulated isobutanol distillers dried grains with solubles and associated fermentation metabolites when fed to male Ross 708 broiler chickens (*Gallus domesticus*)

**DOI:** 10.1371/journal.pone.0219016

**Published:** 2019-07-08

**Authors:** Jason M. Roper, Brenda L. Smith, Jessica M. Caverly Rae, Emily Huang, Carl A. Walker, James L. McNaughton, Alice Chen, Kathy S. Reidinger

**Affiliations:** 1 DuPont Haskell Global Center for Health Sciences, Newark, Delaware, United States of America; 2 DuPont Pioneer, Johnston, Iowa, United States of America; 3 AHPharma, Hebron, Maryland, United States of America; 4 DuPont Industrial Biosciences, Palo Alto, California, United States of America; 5 Butamax Advanced Biofuels, LLC, Wilmington, Delaware, United States of America; University of Illinois, UNITED STATES

## Abstract

*Saccharomyces cerevisiae* genetically engineered to enhance butanol production will be used in a manufacturing process similar to that of fuel ethanol production, including co-production of distillers products for animal feed. A poultry feeding trial was conducted with simulated isobutanol-derived dried distillers grains with solubles (bDDGS), comprising non-fermentable corn solids and heat-inactivated Butamax modified yeast (BMY), to determine potential health effects. Simulated dried distillers grains were produced in 2 variants: bDDGS containing 10% (B10) or 50% (B50) BMY. The BMY concentrations were selected based on a conservative estimate from ethanol-derived distillers grains (eDDGS) approximating 2.5 and 12-fold margins of exposure. The B10 and B50 DDGS were evaluated in a 42-day feeding trial using male Ross 708 broiler chickens fed diets containing eDDGS, B50 DDGS, or B10 DDGS without or with isobutanol, 2,3-butanediol, and isobutyric acid metabolites each at target concentrations of 2 (B10-2), 5 (B10-5), or 10 (B10-10) times the anticipated specification limit in the commercial product. Diets were fed (n = 50 broilers/treatment) in 3 phases: starter phase with 8% DDGS and grower and finisher phases each with 15% DDGS. No statistically significant differences or diet-related effects on mortality, clinical pathology, or organ weights, and no microscopic observations associated with consumption of diets containing B10, B50, or B10 supplemented with metabolites at any targeted exposure level were observed. A lower (*P* < 0.05) mean absolute bursa of Fabricius weight in the B10-10 group compared to the B10 group was considered to be within the range of biological variability. A non-significant trend toward lower weight, gains, and feed intake, and higher feed:gain ratio was observed in the B10-10 group, and was considered a non-adverse palatability effect of consuming high concentrations of metabolites. These results demonstrate that consumption of phase diets containing simulated DDGS from a novel isobutanol production process was well-tolerated.

## Introduction

The ability to produce isobutanol from renewable sources like corn is important because isobutanol is a high value biofuel that can be blended at higher concentrations than existing biofuels [1], helping reduce greenhouse gas emissions without compromising performance. “Bio-isobutanol,” which can be produced in modified ethanol facilities, is also an important renewable chemical used in paints and coatings and as a precursor to isobutylene, from which other high value materials like high octane fuels [2] and synthetic rubber are produced. Bio-based production of isobutanol reduces dependence on fossil sources and provides a direct route to isobutanol production compared with traditional chemical synthesis processes [3, 4]. Publications have supported the biosynthesis of isobutanol from genetically engineered microorganisms such as *Saccharomyces cerevisiae* (SC) [5–9]. The biorefinery process to produce isobutanol from SC is expected to be similar to that of fuel ethanol production, including the co-production of distillers grains products for animal feed, which will contain inactivated SC. Distillers grains containing inactivated yeast, including those produced from genetically engineered SC [10], have an established history of safe use as animal feed ingredients [11, 12]. Distillers grains products from yeast optimized to produce isobutanol are anticipated to have a similar safety profile as the manufacturing process is similar, the base SC strain has a history of use in commercial ethanol production, and the introduced genetic material meets recognized safety criteria, i.e., genetic sequences are limited in size, well characterized, poorly mobilizable, and do not code for toxins, antibiotic resistance, or pathogenic traits [13, 14].

*S*. *cerevisiae* naturally produces isobutanol at low concentrations (0.2 mg/g glucose) [15], which is not an economically viable route unless the yeast has been genetically engineered to increase isobutanol yield. As genetic engineering may alter the metabolite profile, an analysis of metabolites produced by the Butamax modified yeast strain (BMY) was conducted. Thirteen metabolites native to SC were identified, with varying levels compared to wild-type SC. Five of the metabolites, acetic acid, citric acid, lactic acid, succinic acid, and glycerin, are permitted by the US Food and Drug Administration for use in feed at levels not exceeding good feeding practices [16]. Two of the metabolites, ethanol and glucose, are well-defined in traditional ethanol-producing yeasts, as SC is used for ethanol production and glucose is a common carbon source and metabolite in SC [17]. The remaining six metabolites were isobutanol, 2,3-butanediol, isobutyric acid, dihydroxyisovalerate + 2,3-dihydroxy-2-methylbutyrate, alpha-ketoisovalerate, and pyruvate/pyruvic acid, of which the latter three are involved in basic metabolic pathways or evidence indicates they are conserved endogenous metabolites in cattle, swine, and poultry, or common to yeast and plants consumed by these species [18–23]. The genetic, genomic, physiological, biochemical, and metabolic similarities among mammalian species exceed the differences [24]; therefore, use of animal surrogates in toxicity studies is an accepted practice [25], as is the use of surrogate or analogous substances in safety assessment [26]. Previously published studies with ruminants, swine, or an appropriate surrogate species have affirmed the safe consumption of isobutanol, 2,3-butanediol and isobutyric acid for ruminants or swine [27–34]. Additionally, the FDA has approved the use of these 3 metabolites, or closely related chemical surrogates, in feed for ruminants and swine [35–37]. However, based on a comprehensive literature review, a data gap was identified regarding the safety of these 3 metabolites when consumed by poultry. A review of available hazard data from tests in standard laboratory species revealed that isobutanol and isobutyric acid have acute toxicity potential at high doses via the oral and dermal routes of exposure. All three metabolites have the potential to produce irritation based on the results of standardized dermal and ocular irritation screening tests [38], and exposure of rats to aerosols of isobutyric acid produced irritation of the upper respiratory tract [39–40]. Information regarding potential effects of repeated exposure was limited, and included two subchronic studies in rodents administered isobutanol orally via gastric intubation [38] or in a drinking water carrier [41]. Adverse effects were not reported in either study; non-adverse effects observed at dosages ≥ 1000 mg/kg/day were limited to hypoactivity and associated clinical signs of intoxication, transient lower body weights and food consumption, and slight differences in clinical chemistry parameters. In the absence of specific data for isobutyric acid, the isobutanol data were considered relevant for assessment of potential effects, as isobutyric acid is the primary metabolite of isobutanol in humans [42] and rodents (unpublished data from Poet et. al., 2003 as cited in [43]). No relevant repeated-exposure data was identified for 2,3-Butanediol. To address the identified data gap for poultry, a comprehensive feeding trial was conducted in broiler chickens using simulated DDGS and these 3 metabolites. The objectives of the trial were to determine the nutrient composition, including nitrogen-adjusted apparent metabolizable energy (AME_N_) content and amino acid (AA) and phosphorus (P) digestibilities, of simulated isobutanol DDGS products from the proprietary production process, and to identify potential health effects in male broiler chickens fed diets containing these DDGS products or diets containing DDGS products and isobutanol, 2,3-butanediol, and isobutyric acid in combination, each at target concentrations of 2, 5, or 10 times the anticipated specification limit in the commercial bDDGS product. The selections of the eDDGS control group, and target exposure multiples of 2, 5 and 10 for the fermentation metabolites were based on regulatory scientific opinions and technical guidance for the design and conduct of tolerance studies in target species [44–45], and the safety assessment of feed additives for target species [46].

## Materials and methods

The trial was conducted in general accordance with guidance published by the European Food Safety Authority on the design and conduct of tolerance studies [44], and the assessment of the safety of feed additives for the target species [46] and was intended to support the safety of potentially higher levels of these metabolites in poultry fed DDGS derived from isobutanol production. All animal care, housing, and handling conformed to the practices referenced in the Guide for the Care and Use of Agricultural Animals in Agricultural Research and Teaching [47] and were approved by the DuPont Pioneer and testing facility animal care and use committees. Studies were conducted with male Ross 708 broiler chicks (*G*. *domesticus*) obtained from MountAire Hatchery (Princess Anne, MD), and were performed at AHPharma, Inc. facilities (AHP; Hebron, MD). Broilers were housed and managed under conditions similar to those of commercial poultry production.

### DDGS sources

Dried distillers grains with solubles containing 10% (B10) or 50% (B50) heat-inactivated Butamax Modified Yeast product (BMY) were produced from a simulated isobutanol production process which included the typical partial removal of corn oil (Butamax Advanced Biofuels LLC, Wilmington, DE), and commercially-produced ethanol DDGS (eDDGS) were purchased from a US commercial fuel ethanol plant. Commercially produced ethanol DDGS have been estimated to contain 3.9% heat-inactivated yeast [48]. All DDGS materials were stored frozen (approximately -20° C) until used for diet manufacture.

### Determination of AME_N_, amino acid and phosphorus digestibility

Broiler chicks were obtained on the day of hatch (d 0) in sufficient numbers to ensure availability of 480 healthy chicks for evaluating the DDGS sources. Chicks were immediately allocated to 1.219 m x 3.048 m floor pens (40 broilers per pen) with approximately 0.305 m^2^ of available floor space per broiler for 19 d. Pens were equipped with 2 nipple drinkers and floor feeders, and litter consisted of fresh pine shavings with sawdust mixture. A common starter mash diet (22% CP and 3,135 kcal ME/kg diet) was fed from d 0 to d 21. Broilers were not replaced during the acclimation period, nor during the treatment administration period (d 19 to 21). Broilers were weighed and wing-banded on d 19 and allocated randomly into 45.7 cm x 61.0 cm battery cages for a 2-d acclimation period; cages were separate and did not touch any other cage. This evaluation was designed as a randomized complete block with cages within each block assigned randomly and independently to treatments. Four treatments were prepared: eDDGS, B10, or B50 DDGS sources, each mixed in a ratio of 40:60 (wt/wt) with the same commercially available corn hybrid, and the corn hybrid by itself. There were 8 broilers per cage with 12 cages per treatment. Differences in mean body weight (BW) across treatment groups were within 1 SD. On d 21, the common starter diet was removed and broilers were fasted for 6 h after which they were fed only their respective treatment for 6 h; prior work at this facility had determined that a 6 h fasting period was sufficient time to clear fecal material from the gastrointestinal tract. A quantitative collection of excreta was made into clean collection pans under each cage during the 6-h feeding period and for 12 h after the test source was removed; no other feed was provided to the broilers after treatment removal, but water was available. Fecal samples collected from each cage were oven-dried at approximately 100° C for 24 h, or until fecal matter was completely dry. Samples of treatments and feces were submitted for select nutrient analyses. Intakes and fecal weights, along with feed and fecal analyzed nutrients, were converted to a DM basis for all calculations. Initial AME and AME_N_ values of all treatments (corn and blended DDGS:corn sources) were calculated as: AME (kcal/kg DM) = [(Intake GE–Fecal GE) / DM Intake, g] x 1000; AME_N_ (kcal/kg DM) = [AME–(8.730 x ((Intake N–Fecal N) / DM Intake, g))] x 1000; where GE is gross energy content in calories, N is nitrogen content in g, and 8.730 is the nitrogen correction factor [49]. The final AME_N_ value of each DDGS source was calculated as DDGS AME_N_ (kcal/kg DM) = [blended DDGS:corn source AME_N_−(corn hybrid AME_N_ x 0.6)] / 0.4, where AME_N_ is the energy content in kcal/kg DM, and 0.4 and 0.6 are the proportions of DDGS and corn, respectively, in the blended treatment. Individual AA and P digestibility was calculated as Digestibility (%) **=** [(Intake AA or P–Fecal AA or P) / Intake AA or P)] x 100, where AA is the individual amino acid in g, and P is P content in g.

### 42-Day feeding study

#### Experimental design

Broiler chicks were obtained on the day of hatch (d 0) in sufficient numbers to ensure availability of 300 healthy chicks for the conduct of the study. Housing conditions and management were as previously described [50], except for pen litter which consisted of new pine shavings with a minimal amount of saw dust. The study was designed as a randomized complete block with 6 treatments: a control treatment prepared with eDDGS; treatments prepared with B10 or B50 DDGS sources; and 3 additional treatment diets prepared with the B10 DDGS source and supplemented with the fermentation metabolites isobutanol, 2,3-butanediol and isobutyric acid, each at target concentrations representing 2, 5, or 10 times (B10-2, B10-5, and B10-10, respectively) the anticipated specification limit rates of 100 mg/kg isobutanol, 13,000 mg/kg 2,3-butanediol, and 6,000 mg/kg isobutyric acid in the final commercial product. Broilers were allocated to pens (10 per pen) and pens to treatment groups (5 pens per treatment). Differences in mean BW across treatment groups were within 1 SD. In case of mortality, broilers were not replaced during the conduct of the study.

#### Diet preparation and metabolite addition

Treatment diets were offered fresh weekly as mash feed for *ad libitum* consumption (except as noted) and were fed in 3 phases: starter (d 0 to 21), grower (d 22 to 35), and finisher (d 36 to 42). All diets were formulated using composition analyses, and in consideration of the AME_N_ results determined for the eDDGS, B10, and B50 sources (Table 1). Incorporation rates of 8 and 15% DDGS, recommended by previous researchers as having no negative effects on performance or meat quality measures [51–54], were utilized in starter and grower-finisher phases, respectively. Each phase diet was formulated, using National Research Council (NRC) recommendations as a guideline [55], to closely approximate the nutrient requirements of a commercial broiler (Table 2). Inclusion of DDGS was equalized across treatments within each phase, and protein sources, crystalline amino acids, and minerals were added in the amounts necessary to meet requirements for protein, lysine, methionine (Met), cystine (Cys), calcium (Ca), and P as per the formulation criteria. Diets within a growth stage were formulated to be isocaloric; in order to formulate diets to be isonitrogenous it was necessary to lower the targeted crude protein (CP) values for starter and grower phases due to the high CP content of the B50 source. Diets did not contain coccidiostats or antibiotics, and a minimum of 1% fat was specified for dust control.

**Table 1:** Analyzed nutrient composition^1^^,^^2^ (as-fed basis) of DDGS sources.

Item	eDDGS	B10 DDGS	B50 DDGS
Proximates, energy, and minerals (% except as noted)			
DM	86.6	88.5	89.1
CP	25.4	27.0	39.3
Crude fat	11.4	5.29	6.18
Gross energy, kcal/kg	4,402	4,367	4,571
AME_N_, kcal/kg^3^	2,175	2,179	2,376
AME_N_, % of gross energy^4^	54.10	54.72	55.26
Crude fiber	7.04	10.1	5.46
Ash	5.27	1.11	3.41
Calcium	0.0392	0.0135	0.0119
Total phosphorus	1.04 (87.18^a^, 4.46)	0.262 (67.39^b^, 12.63)	0.811 (85.04^a^, 3.89)
Essential amino acid, %			
Arg	1.15 (93.23^b^, 1.52)	1.11 (92.84^b^, 1.73)	1.63 (95.78^a^, 0.89)
His	0.737 (92.99^b^, 1.50)	0.717 (92.56^b^, 1.73)	0.897 (94.88^a^, 1.06)
Ile	0.946 (89.73^b^, 2.32)	1.02 (90.25^b^, 2.45)	1.62 (94.42^a^, 1.26)
Leu	2.90 (93.85^b^, 1.44)	3.23 (94.43^ab^, 1.46)	3.34 (95.50^a^, 0.94)
Lys	0.834 (89.27^b^, 2.84)	0.947 (89.93^b^, 3.19)	2.09 (95.69^a^, 1.02)
Met	0.417 (93.49^b^, 1.40)	0.634 (94.38^ab^, 1.08)	0.729 (95.65^a^, 0.88)
Phe	1.27 (92.46^b^, 1.71)	1.41 (92.79^b^, 1.84)	1.78 (95.16^a^, 1.07)
Thr	1.05 (86.81^b^, 2.52)	1.11 (87.30^b^, 2.58)	1.69 (92.70^a^, 1.32)
Trp	0.158 (89.30^b^, 2.85)	0.172 (89.76^b^, 2.49)	0.348 (94.15^a^, 1.12)
Val	1.22 (89.59^b^, 2.23)	1.30 (90.01^b^, 2.38)	1.94 (94.01^a^, 1.20)
Non-essential amino acid, %			
Ala	1.67 (91.94^b^, 1.68)	1.87 (92.53^ab^, 1.72)	2.32 (94.75^a^, 0.99)
Asp	1.69 (89.79^b^, 2.31)	1.81 (90.18^b^, 2.51)	3.29 (94.70^a^, 1.12)
Cys	0.380 (89.44^a^, 3.49)	0.494 (89.12^a^, 2.45)	0.414 (90.25^a^, 1.82)
Glu	4.62 (93.56^b^, 1.46)	4.87 (93.89^ab^, 1.54)	5.46 (95.27^a^, 0.93)
Gly	1.12 (84.57^b^, 2.54)	1.09 (83.94^b^, 3.08)	1.63 (90.61^a^, 1.49)
Pro	2.14 (92.38^b^, 1.51)	2.22 (92.68^a^, 1.66)	2.09 (93.72^a^, 1.06)
Ser	1.30 (90.60^b^, 2.00)	1.36 (90.82^b^, 1.97)	1.74 (93.84^a^, 1.12)
Tyr	0.874 (90.82^b^, 2.06)	0.899 (90.95^b^, 2.45)	1.14 (94.38^a^, 1.29)

^1^All values except AME_N_ and nutrient digestibility represent the mean of 2 samples; n = 12 for AME_N_ and nutrient digestibility. The DDGS AME_N_ values were calculated from the respective DDGS:corn mixture AME_N_ mean and corn source AME_N_ mean; as such, statistical analysis was not performed on these AME_N_ and AME_N_ % of gross energy values.

^2^Parenthetical values represent determined nutrient digestibility % and SD of the corn:DDGS mixtures; values with unlike superscripts differ P < 0.05.

^3^AME_N_ value of DDGS source converted to an as-fed basis based on dry matter determined within the AME_N_ trial (eDDGS = 90.2%, B10 = 89.7%, B50 = 91.9%).

^4^AME_N_ digestibility as a % of gross energy is calculated based on gross energy measurements determined within the AME_N_ trial (eDDGS = 4,021 kcal/kg, B10 = 3,980 kcal/kg, B50 = 4,300 kcal/kg).

**Table 2:** Ingredient and targeted nutrient concentrations of phase bulk basal diets.

Item	Starter (d 0 to 21)			Grower (d 22 to 35)			Finisher (d 36 to 42)		
	eDDGS	B10	B50	eDDGS	B10	B50	eDDGS	B10	B50
Ingredient, %										
Corn	49.55	49.80	53.72	46.97	47.45	54.79	49.08	49.57	56.91	
Soybean meal	31.53	31.25	28.53	25.75	25.22	20.12	23.28	22.75	17.65	
DDGS	8.00	8.00	8.00	15.00	15.00	15.00	15.00	15.00	15.00	
Soybean oil	6.03	6.00	4.77	7.95	7.89	5.58	8.57	8.51	6.21	
Salt	0.42	0.41	0.46	0.33	0.33	0.41	0.28	0.28	0.36	
Limestone	1.68	1.65	1.70	1.43	1.37	1.46	1.38	1.32	1.41	
Di-Cal	1.59	1.70	1.64	1.38	1.60	1.48	1.24	1.46	1.34	
Choline chloride	0.027	0.027	0.028	0.025	0.025	0.025	0.026	0.027	0.027	
Poultry VTM^1^	0.62	0.62	0.62	0.63	0.63	0.62	0.63	0.62	0.62	
DL Met	0.47	0.45	0.47	0.39	0.34	0.39	0.34	0.30	0.34	
L-Lys HCl	0.080	0.078	0.057	0.16	0.15	0.11	0.17	0.17	0.13	
Targeted nutrient guidelines by diet phase (% except as noted)^2^										
ME, kcal/kg	3,031			3,086			3,142			
CP	20.0			19.0			18.0			
Calcium	1.05			0.90			0.85			
Total phosphorus	0.74 to 0.78 (0.70)			0.69 to 0.76 (0.65)			0.64 to 0.72 (0.60)			
Available phosphorus	0.45			0.42			0.39			
Lys	1.20			1.14			1.08			
Met	0.50			0.45			0.40			
Met + Cys	1.02			0.92			0.85			
Arg	1.15 to 1.20 (1.25)			1.01 to 1.09 (1.10)			0.94 to 1.02 (1.00)			
Thr	0.79 to 0.80 (0.80)			0.75 to 0.76 (0.74)			0.71 to 0.72 (0.68)			
Trp	0.20			0.18			0.16 to 0.17 (0.16)			
Sodium	0.20			0.18			0.16			
Choline, g/kg	1.35			1.25			1.25			

^1^Vitamin-mineral premix supplied (minimum) per kg diet: selenium, 0.3 mg; vitamin A, 1,703 IU; vitamin D_3_, 568 ICU; vitamin E, 3.7 IU; menadione, 0.2 mg; vitamin B_12_, 0.002 mg; biotin, 0.01 mg; choline, 92 mg; folic acid, 0.3 mg; niacin, 8.5 mg; pantothenic acid, 2.3 mg; pyridoxine, 0.2 mg; riboflavin, 1.1 mg; and thiamine, 0.3 mg).

^2^Where ranges are noted, nutrient guideline target values varied for the individual diets, based on compositional differences in protein, amino acid, and mineral concentrations among the eDDGS, B10, and B50 sources; values in () represent NRC values for reference.

Bulk basal diets for all phases were manufactured at the DuPont Pioneer Livestock Nutrition Center (Polk City, IA). The B10 bulk basal phase diets were prepared in a Sudenga M2000 ribbon mixer, and the eDDGS and B50 bulk basal phase diets were prepared in that order using a Sudenga M500 ribbon mixer (Sudenga Industries, Inc.; George, IA). Mixers were cleaned before and after each diet manufacture by flushing with ground corn and cleaning with vacuum and compressed air. Diets were packaged and held in cold storage until being shipped refrigerated to AHP, where they were stored refrigerated upon receipt. Preparation of the treatment phase diets from the eDDGS, B10 and B50 bulk phase diets was performed by AHP near the initiation of feeding for each respective phase. Each B10 bulk basal phase diet was subdivided for metabolite addition at the respective target concentrations. Isobutanol and isobutyric acid were obtained from Sigma Aldrich (St. Louis, MO), and 2,3-butanediol was obtained from EMD Millipore Corporation (Billerica, MA). Treatment diets were prepared in the order of eDDGS, B50, B10, B10-2, B10-5, and B10-10 to minimize the potential for cross-contamination. Because metabolites in liquid form were added to the latter 3 treatments, moisture content was balanced among all treatment phase diets using water, with the total quantity of supplemental water and/or metabolites added to any individual diet being equal to the total quantity of metabolites added to the B10-10 treatment phase diet. Briefly, an appropriate quantity of bulk phase diet was added to a Sudenga M1000 ribbon mixer. The metabolites, and water (if required or by itself), were mixed in a secondary container, added to the bulk phase diet, and mixed for approximately 10 minutes. The mixer was cleaned before and after each diet manufacture by flushing with ground corn and cleaning with compressed air. Following completion of mixing and sample collection, finished treatment phase diets were packaged and stored refrigerated until fed.

Composite samples of eDDGS, B50, and B10 treatments were prepared at treatment phase diet manufacture and at the end of each phase following 1 week under ambient conditions during the last week of the phase. Both sample sets were submitted for nutrient analyses. Isobutanol, 2,3-butanediol and isobutyric acid concentrations were determined in composite samples of eDDGS, B50, and B10 treatments prepared at the time of treatment phase diet manufacture. Metabolite concentrations in B10-2, B10-5, and B10-10 treatment phase diets were evaluated to determine homogeneous distribution using individual samples collected at the beginning, middle, and end of each diet manufacture; mean concentration was also used to calculate analyzed value as a percent of target value. From previous experience with diets containing comparable metabolite concentrations, it was known prior to study initiation that metabolite loss would occur during the diet mixing, storage, and feeding processes due to the effect of exposure temperature on vapor pressures of the metabolites. Thus, samples of B10-2, B10-5, and B10-10 treatment phase diets were collected during the feeding period to determine metabolite concentrations under 1) ambient conditions and 2) refrigerated storage. Treatment samples prepared concurrently with the addition of fresh feed to the feeders for that week were maintained open in the trial room at the start of each week for 7 days, then stored; these samples were collected on d 7, 14, 21, 28, 35, and 42. For evaluation of metabolite concentration under refrigerated storage, samples were prepared concurrently with the fresh feed addition for each week on d 0, 7, 14, 21, 28, and 35. Diet samples for nutrient or metabolite analyses were stored frozen (approximately -20°C) immediately after collection, and shipped frozen to the designated laboratories for the respective analyses.

#### Growth performance measures and metabolite intakes

Following determination of individual BW at day 0, BW and feed weights (including amount of feed added and amount remaining by pen) were determined every 7 days with BW gain (BWG), feed intake and feed conversion (uncorrected and mortality-corrected feed:gain ratios) calculated weekly and overall (days 0 to 42). Uncorrected feed:gain was calculated as g of feed intake per g of BW gain; mortality-corrected feed:gain was calculated by adding mortality BW at removal to the live weight of broilers remaining in a pen. The average consumed doses of isobutanol, 2,3-butanediol, and isobutyric acid were calculated weekly for each group as Metabolite intake (mg/kg BW/day) = (C x ADFI_ind_) / BW, where C = the average weekly concentration of metabolites in each respective diet (mg/kg diet) calculated by averaging the measured concentrations determined at the start and end of each week of feeding; ADFI_ind_ = ADFI (Average Daily Feed Intake; g diet/broiler/day) calculated as the sum of the ADFI for each replicate divided by the number of surviving broilers at the end of each week; and BW = average weekly broiler BW (g/broiler) calculated for each group by averaging the BW at the start and end of each week.

#### Blood and tissue collection

For endpoints measured on an individual basis, where animals are housed socially and the experimental unit is the pen, it was determined from a prospective power analysis that a minimum of 4 pens with 5 individual animals per pen are required to detect an approximate effect size of the SD among individuals for each endpoint with at least 80% power in a study with 6 treatment groups. Thus, 5 broilers per pen (25 total per treatment) were randomly selected for post-mortem evaluation of clinical and anatomic pathology endpoints utilizing blood and tissue samples collected at the end of the 42-d feeding period. All surviving broilers were fasted for a minimum of 8 h (but no more than 12 h) prior to collection. Broilers from all treatment groups within a block were sampled and necropsied on the same day of scheduled collection to process an equal number of broilers from each treatment on each day. Whole blood samples were collected via cardiac puncture and dispensed into vacutainer tubes containing lithium heparin for hematology analysis (2.0 mL) and duplicate microtainer capillary tubes for serum chemistry analysis (0.5 mL/tube). All tubes were stored on ice prior to shipment for same-day delivery and immediate analysis at Antech Diagnostics (Memphis, TN). Broilers were humanely euthanized by cervical dislocation following blood collection and a complete necropsy was conducted. The following tissues were collected: brain, liver, kidney, heart, bursa of Fabricius, lungs, testes, crop (each weighed, with paired organs or tissues weighed together); skeletal muscle (1 minor pectoralis), adrenal glands, thyroid glands, eyes, esophagus, proventriculus, ventriculus, duodenal loop (after pancreas removal), ileum (from Merkel’s diverticulum to the ileo-caecal junction), left-lateral cecum, pancreas, thymus, trachea and spleen. All tissues were placed into 10% neutral-buffered formalin, except for the eyes which were placed in Davidson’s total fixative. The lungs were evaluated microscopically based on the previously-described irritation potential of the fermentation metabolites identified during the literature review. Consistent with published technical guidance for the safety assessment of feed additives for the target species [46], the kidneys, skeletal muscle, and bursa of Fabricius were evaluated microscopically (DuPont Haskell, Newark, DE) based on observed differences in clinical and anatomic endpoints potentially associated with these tissues, as described in the results. Remaining broilers not selected for post-mortem sample collections were humanely euthanized as described previously. Carcasses of all broilers were disposed of via composting according to local regulations.

### Chemical analyses

Duplicate samples of eDDGS and B10 and B50 DDGS sources were evaluated for nutrient composition and mycotoxin profile at EPL Bio Analytical Services (Niantic, IL). Dry matter, ash, Ca and P, protein, and crude fiber analyses were performed according to AOAC International methods [56–58]. Fat was analyzed according to AOCS methods [59], and gross energy (GE) content was determined using a bomb calorimeter. Amino acid analyses were performed as follows: Trp was determined as per Rogers and Pesti [60], with modifications to use reverse phase ultra-performance liquid chromatography (UPLC) with ultraviolet detection (UV); Cys and Met were analyzed as cysteic acid and methionine sulfone, respectively [56] using reverse-phase UPLC with ultraviolet detection [61, 62]; all other reported AAs were determined using reverse-phase UPLC with UV detection [61, 62]. Aflatoxins, T-2 toxin, and ochratoxin were determined using multitoxin UPLC-MS/MS, and fumonisins, deoxynivalenol, 3- and 15-acetyl deoxynivalenol, and zearalenone were analyzed using multitoxin UPLC-MS/MS [63]. Treatment and fecal samples collected for AME_N_ and digestibility determinations were analyzed at AHP (Hebron, MD) for dry matter (DM) (100°C for 24 h) and GE using a bomb calorimeter with benzoic acid as an internal standard, and at EPL Bio Analytical Services for CP, P, and AA, all as previously described. Diet samples of eDDGS, B50, and B10 phase treatments collected during the feeding study were evaluated for proximate composition, minerals (Ca and P), GE, and AA at EPL Bio Analytical Services, as described. Selected nutrients critical for poultry production, and their associated 90% confidence interval (CI) values were compared with feeding program nutrient guidelines and recommended requirements [55]. The 90% CI were calculated as CI = targeted nutrient value ± t x (targeted nutrient value x 10%), where t = 1.645 (z-value for 90% CI), and 10% is the assay CV provided by the analytical laboratory. Analytical results of samples collected at the start and end of each feeding phase were compared to evaluate nutritional stability during the feeding period. Diet samples for metabolite analyses were weighed, extracted, diluted and analyzed at Critical Path Services (Garnet Valley, PA) using a Thermo Scientific Trace GC Ultra gas chromatography system coupled with a Thermo Scientific DSQ II mass spectrometer (Thermo Electron Corporation, Austin, TX). A 6-point, weighted linear calibration curve for each metabolite was used for quantification, and metabolite concentrations were calculated as: metabolite (mg/kg diet) = (extract metabolite μg/mL × extract volume mL × dilution factor) / sample weight, g.

### Hematology and serum analyses

Hematology analyses included total white blood cell (WBC) count, hematocrit, and absolute and percent differentials for lymphocytes, monocytes, heterophils, eosinophils, bands and basophils. The WBC count and hematocrit were performed manually. Whole blood samples were centrifuged at 4200 rpm for 5 min prior to immediate chemistry analysis. Total protein, glucose, albumin, globulin, aspartate aminotransferase, alanine aminotransferase, alkaline phosphatase, gamma glutamyl-transpeptidase, total bilirubin, urea nitrogen (BUN), creatinine, P, Ca, magnesium, sodium, potassium, chloride, cholesterol, triglycerides, amylase, lipase, and creatine phosphokinase were measured in serum using an AU5400 Chemistry Analyzer (Beckman Coulter, Brea, CA). Albumin:globulin, BUN:creatinine, and sodium:potassium ratios were calculated from the respective results.

### Statistical analyses

For the AME_N_ trial, mean values for each treatment and data parameter were analyzed using a mixed model analysis of variance (ANOVA) and multiple comparison procedure. Differences were considered significant if the P value was < 0.05. Fisher’s least significant difference (LSD) procedure was used to discriminate among the means.

The primary comparisons of interest in the 42-day feeding trial were 1) eDDGS control versus each remaining treatment group, and 2) B10 versus B50 and each level of metabolite addition (B10-2, B10-5, and B10-10). The statistical models used depended on the characteristics of each endpoint. Feed consumption and adjusted feed gain ratio data were collected or calculated on a per pen basis. These endpoints were modeled considering cage to be the unit of replication (experimental unit) and the unit of observations. Data for BW, hematology, coagulation, clinical chemistry, and absolute and relative organ weights were collected or calculated on an individual broiler basis. These endpoints were modeled considering cage to be the unit of replication and broiler to be the unit of observation. For all continuous endpoints, if < 50% of non-missing data values were at a uniform value, then mixed model analysis was applied. Otherwise, statistical analysis was not performed.

The following linear mixed model was used for endpoints measured on a pen basis: *Y*_*ij*_ = *μ*+*α*_*i*_+*β*_*j*_+*ε*_*ij*_, where Y_*ij*_ = observed pen response fed diet *i* in block *j*, *μ*, = overall mean, *α*_*i*_ = treatment effect, *β*_*j*_ = random block effect, and *ε*_*ij*_, residual error. This model assumed that random effects *β*_*j*_ and *ε*_*ij*_ were independent of each other and that $\beta_{j}∼iid\;N(0,\sigma_{b}^{2}),\varepsilon_{ij}∼iid\;N(0,\sigma_{e}^{2})$. For endpoints determined on an individual broiler basis, the following linear mixed model was used: *Y*_*ijk*_ = *μ*+*α*_*i*_+*β*_*j*_+*γ*_*ij*_+*ε*_*ijk*_, where *Y*_*ijk*_ = response from broiler *k* fed diet *i* in block *j*, *μ* = overall mean, *α*_*i*_ = treatment effect, *β*_*j*_ = random block effect, *γ*_*ij*_ = the effect of pen fed treatment *i* in block *j*, and *ε*_*ijk*_ = residual error, and *k* = 5 for pathology endpoints and ≤ 10 for in-life data. This model assumed that random effect *β*_*j*_ was independent of *γ*_*ij*_ and *ε*_*ijk*_; the random effect of block *β*_*j*_ was assumed to follow a normal distribution $\beta_{j}∼iid\;N(0,\sigma_{b}^{2})$. A compound symmetry covariance structure was used for the variance covariance matrix of the remaining 2 random terms, pen and broiler, to allow positive or negative covariance among individual broilers within the same pen. Data were analyzed using the PROC MIXED procedure (SAS version 9.4 software, SAS Institute Inc., Cary, NC). Means and 95% CI of the means [64] were estimated from the model. The normality and homogeneous variance assumptions were evaluated by examination of the normal probability plots of studentized residuals and scatter plots of studentized residuals by predicted values, for each endpoint subjected to mixed-model analysis. Where the assumptions were questionable, transformations (logarithm, square, etc.) were evaluated as remedial measures. To facilitate consistency and clarity in interpretation of analysis results for endpoints measured on a weekly basis, transformations were applied only when the same transformation was appropriate for all measurement intervals. The normality and homogeneous variance assumptions were rechecked after transformation. When transformations were applied, means and CI were reported after back-transformation. For mortality data, Fisher’s exact test was conducted using the PROC FREQ procedure. The false discovery rate (FDR) method [65, 66] was applied as a post-hoc procedure to account for multiplicity due to the large number of endpoints evaluated in this study, and *P* values were adjusted accordingly. The FDR adjustment was made to the *P* values across all endpoints within each pairwise comparison between diet groups and differences were considered significant if the FDR adjusted *P* value was < 0.05.

## Results and discussion

### DDGS characterization

With few exceptions, the analyzed nutrient compositions of the DDGS sources (Table 1) were similar to those determined by others [67–75]. The protein content of the B50 source was approximately 12 to 14 points higher than that of B10 DDGS and eDDGS sources, respectively, and concentrations of amino acids Ile, Lys, Met, Phe, Trp, Val, Asp, and Gly were also much higher. The increased protein and AA content likely reflects the higher yeast inclusion for the B50 DDGS source, as yeast contributes approximately 20% to the composition of the DDGS amino acids [76]. Calculated AME_N_ values were similar for eDDGS and B10 DDGS sources, but were approximately 200 kcal/kg higher for the B50 DDGS. AME_N_ content and digestibility were similar to that of DDGS sources evaluated by [71, 72]. The ash and P values for B10 were low. Phosphorus content is known to be variable among DDGS sources and can be influenced by the P content in the corn grain source and by processing conditions such as starch fermentation and solubles addition [77]. Mycotoxin occurrence in the DDGS sources was limited (S1 Table). Fumonisins and deoxynivalenol were well below established guideline values of 50 and 10 mg/kg, respectively [78], especially when included at 8% and 15% in the prepared diets. Zearalenone was present in eDDGS, B10 DDGS, and B50 DDGS at 50.9, 262, and 153 ppb, respectively, and below the guidance limit of 3000 ppb in corn by-products [79]; US FDA dietary limits for zearalenone have not been established for broilers. Phosphorus digestibility, as measured in the corn:B10 DDGS mixture, was lower when compared to the corn:eDDGS or corn:B50 DDGS mixtures, and digestibility of most essential amino acids was higher for the corn:B50 DDGS mixture, and similar between corn:eDDGS and corn:B10 DDGS mixtures.

### Analyzed nutrient composition of treatment phase diets

Analyzed nutrient compositions of the start and end samples collected from the individual phase treatment diets are presented in S2, S3 and S4 Tables; a summary of the calculated start and end averages of the phase treatment diets is presented in Table 3. All diets met or exceeded NRC recommendations [55] for most analytes, and were generally within the 90% CI of the nutrient guidelines for those nutrients considered critical for poultry performance, with 2 exceptions. The combined Met + Cys value for the B50 grower phase diet was below the 90% CI (0.77 to 1.07) at the start of the feeding period. This slight, spurious difference did not impact the nutritional value of the diet, because the value exceeded NRC recommendations [55], and the observed value at the end of the feeding period was within the 90% CI. Calcium values for the B50 finisher phase diets at the start and end of that feeding period were below NRC recommendations [55] and the 90% CI (0.71 to 0.99). However, modern broiler chickens may be more efficient at utilizing Ca, and the NRC-recommended values for Ca in both grower and finisher phases may be excessive for measures such as weight gain and feed conversion. It has been concluded that reducing dietary Ca by 15 to 20%, with consideration for Ca:P balance is not detrimental to performance [80], and a similar reduction in Ca has been recommended by the Ross 708 breeder [81, 82]. Importantly, total Ca:total P ratios were maintained at 1.0 to 1.4 across all diets in all phases, and at the start and end of each feeding period, similar to a 1:1 ratio, which was found to maximize BW gain and feed conversion [83]. Overall, the phase diets were considered nutritionally adequate to support production and prevent deficiencies, and there were no biologically-relevant changes in nutrient composition under conditions of use during their respective feeding periods.

**Table 3:** Summarized nutrient composition analyses (as-is basis) of eDDGS, B10, and B50 starter, grower, and finisher phase bulk basal diets^1^.

Item	Starter (d 0 to 21)			Grower (d 22 to 35)			Finisher (d 36 to 42)		
	eDDGS	B10	B50	eDDGS	B10	B50	eDDGS	B10	B50
Proximates, energy, and minerals (% except as noted)									
Moisture	12.5	12.5	12.7	13.2	13.2	13.6	14.0	14.0	13.6
CP	20.6	20.1	20.2	19.4	18.9	18.9	17.8	17.9	18.7
Crude fat	8.33	7.63	7.17	9.66	10.0	7.71	11.1	11.9	8.00
Gross energy, kcal/kg	4100	4110	4000	4125	4170	4090	4215	4215	4100
Crude fiber	2.90	3.08	2.64	2.91	3.61	2.67	2.94	3.52	2.85
Ash	4.96	4.61	4.64	4.64	4.16	4.25	4.19	3.66	3.68
Calcium	0.931	0.949	1.02	0.843	0.854	0.918	0.753	0.755	0.680
Phosphorus	0.784	0.751	0.828	0.795	0.728	0.775	0.703	0.655	0.672
Essential amino acid, %									
Arg	1.20	1.23	1.17	1.09	1.07	1.05	1.01	0.998	0.971
His	0.510	0.538	0.514	0.499	0.485	0.487	0.482	0.453	0.462
Ile	0.816	0.903	0.893	0.793	0.795	0.831	0.753	0.751	0.798
Leu	1.65	1.80	1.74	1.69	1.73	1.69	1.64	1.64	1.64
Lys	1.22	1.19	1.24	1.20	1.13	1.16	1.05	1.05	1.14
Met	0.641	0.621	0.728	0.579	0.559	0.560	0.528	0.539	0.508
Met + Cys	0.926	0.906	1.01	0.838	0.831	0.782	0.797	0.800	0.728
Phe	0.943	1.04	0.977	0.930	0.936	0.939	0.886	0.900	0.883
Thr	0.798	0.801	0.792	0.726	0.731	0.768	0.694	0.696	0.741
Trp	0.212	0.208	0.200	0.179	0.174	0.179	0.172	0.172	0.163
Val	0.885	0.975	0.973	0.879	0.884	0.928	0.842	0.839	0.902
Non-essential amino acid, %									
Ala	0.953	1.04	1.05	0.986	1.02	1.05	0.945	0.946	1.05
Asp	1.90	2.05	2.09	1.80	1.83	1.85	1.66	1.60	1.76
Cys	0.285	0.285	0.288	0.260	0.272	0.222	0.269	0.261	0.220
Glu	3.29	3.66	3.59	3.33	3.37	3.23	3.14	3.05	3.07
Gly	0.788	0.853	0.832	0.787	0.774	0.797	0.746	0.733	0.766
Pro	1.12	1.22	1.16	1.16	1.17	1.13	1.13	1.12	1.10
Ser	0.897	1.02	0.979	0.928	0.928	0.921	0.886	0.874	0.868
Tyr	0.504	0.562	0.534	0.509	0.509	0.520	0.479	0.510	0.501

^1^Values are the calculated average of the start and end values presented by diet phase in S2, S3 and S4 Tables.

### Analyzed metabolite concentrations of treatment phase diets

Targeted and analyzed metabolite concentrations are presented in Tables 4 and 5, respectively. Low concentrations of 2,3-butanediol detected in eDDGS grower and finisher phase diets (Table 5) were not unexpected as these metabolites have been described as normal components of commercial eDDGS [84, 85]. Low concentrations of isobutanol and isobutyric acid were detected in all B10 and B50 phase diets. Their presence was likely due to residual intracellular metabolites trapped in the BMY cells. During preparation, BMY cells were concentrated and washed following fermentation, then processed into dry form. It is likely that the BMY continued to produce intracellular metabolites until heat inactivation during the drying process. Results of the analyses demonstrated that all metabolites were homogenously distributed in all B10-2, B10-5, and B10-10 phase diets, with relative SD values ranging from 0.5% to 8.7%. In general, diets containing the supplemental metabolites were lower than their respective target concentrations (Table 4), although some treatment phase diets did fall within the acceptance criteria of 80 to 120% of target. Metabolite concentration as a percent of target concentration in the B10-2, B10-5, and B10-10 treatment diets and across diet phases ranged from 44% to 82% for isobutanol, 59% to 82% for 2,3-butanediol, and 60% to 82% for isobutyric acid. The low concentrations observed during treatment diet mixing and administration likely resulted from the volatility of the metabolites, the large surface area of the diets, and the ambient temperature range in the trial room. Based on these results, the actual measured concentrations of the metabolite-supplemented diets ranged from 1 to 2 times the anticipated specification limit for B10-2, 3 to 5 times for B10-5, and 6 to 10 times for B10-10. Combined actual metabolite exposures represented multiples of 1, 3 and 6 times the anticipated specification limit for the B10-2, B10-5 and B10-10 groups, respectively. These differences from the intended targets did not impact the overall quality or integrity of the study, as the data are interpreted in the context of actual, rather than target, multiples of the anticipated specification limit. Further, the observed low concentrations compared with target concentrations support the likelihood that these metabolites may be lower than expected in commercial bDDGS fed to poultry due to metabolite losses during storage and handling, and exposure of blended feeds containing bDDGS to ambient conditions.

**Table 4:** Targeted concentrations^1^ (as-is basis) of metabolites added to treatment phase diets prepared using B10 DDGS bulk basal diets.

Metabolite, mg/kg	B10-2	B10-5	B10-10
	Starter (days 0 to 21)		
Isobutanol	65	130	225
2,3-Butanediol	2,080	5,200	10,400
Isobutyric acid	960	2,400	4,800
	Grower (days 22 to 35)		
Isobutanol	95	195	380
2,3-Butanediol	3,900	9,750	19,500
Isobutyric acid	1,800	4,500	9,000
	Finisher (days 36 to 42)		
Isobutanol	90	190	365
2,3-Butanediol	3,900	9,750	19,500
Isobutyric acid	1,800	4,500	9,000

^1^Target concentrations of added metabolites; some diet ingredients had low detectable levels of endogenous metabolites.

**Table 5:** Analyzed metabolite concentrations^1^ in treatment phase diets at the time of preparation.

Metabolite, mg/kg	eDDGS	B10	B50	B10-2	B10-5	B10-10
	Starter Phase (d 0 to 21)					
Isobutanol	<LOD^2^	1.20^3^	3.00^3^	44.1 (2.64)	95.5 (8.32)	172 (8.7)
RSD, %^2^				6.0	8.7	5.1
2,3-Butanediol	<LOD	<LOD	<LOD	1,545 (53.1)	3,876 (260)	8,513 (494)
RSD, %				3.4	6.7	5.8
Isobutyric acid	<LOD	20.3^3^	28.8^3^	637 (30.3)	1,810 (99.0)	3,946 (203)
RSD, %				4.8	5.5	5.1
	Grower Phase (d 22 to 35)					
Isobutanol	<LOD	3.22^3^	6.78^3^	68.7 (3.1)	150 (11.7)	313 (12.2)
RSD, %				4.5	7.8	3.9
2,3-Butanediol	234	<LOD	<LOD	2,663 (128)	6,965 (226)	15,101 (587)
RSD, %				4.8	3.2	3.9
Isobutyric acid	<LOD	56.7^3^	121^3^	1,301 (34.5)	3,289 (116)	7,271 (247)
RSD, %				2.7	3.5	3.4
	Finisher Phase (d 36 to 42)					
Isobutanol	<LOD	2.04^3^	5.80^3^	39.9 (0.6)	120 (4.0)	226 (10.1)
RSD, %				1.6	3.4	4.5
2,3-Butanediol	91.2^3^	<LOD	<LOD	2,305 (13.0)	6,301 (438)	13,138 (590)
RSD, %				0.6	6.9	4.5
Isobutyric acid	<LOD	65.5^3^	120^3^	1,126 (5.3)	2,719 (140)	5,875 (285)
RSD, %				0.5	5.1	4.9

^1^Reported values represent the mean of samples analyzed in duplicate from the start, middle, and end of treatment diet production (n = 6; SD in parentheses).

^2^Relative standard deviation (RSD). Limits of detection (LOD) were 1, 100, and 50 mg/kg for Isobutanol, 2,3-Butanediol and Isobutyric acid, respectively.

^3^Results below the LOD were assigned the LOD value for mean calculation when at least one replicate value exceeded the LOD.

Average isobutanol, 2,3-butanediol and isobutyric acid consumption was calculated weekly based on analyzed dietary metabolite concentrations, ADFI and average weekly BW. The overall mean consumed doses for individual metabolites and all metabolites combined are summarized in Table 6; weekly values are presented in S5 Table. Based on the differences in concentration for the specification limits of isobutanol, 2,3-butanediol and isobutyric acid in isobutanol DDGS, and in consideration of the target multiples of 2, 5 and 10 times the commercial specification, 2,3-butanediol contributed most significantly to the overall metabolite exposure, followed by isobutyric acid and then isobutanol. The no-observed-adverse-effect level for metabolite consumption was represented by the B10-10 group, with consumed metabolite intakes being 15.4, 1,318, and 539 mg/kg BW/day for isobutanol, 2,3-butanediol and isobutyric acid, respectively, and a total consumed metabolite intake of 1,872 mg/kg BW/day. These intakes correspond to relative incorporations of 10, 7, and 6 times the anticipated specification limits for isobutanol, 2,3-butanediol and isobutyric acid, respectively.

**Table 6:** Overall metabolite concentrations in treatment diets fed to broilers and calculated metabolite intakes.

Item	eDDGS	B10	B50	B10-2	B10-5	B10-10
Overall metabolite mean concentration, mg/kg						
Isobutanol	<LOD	2.0	4.7	25.9	63.0	124
2,3 Butanediol	103	<LOD	<LOD	1,724	4,861	10,469
Isobutyric acid	<LOD	40	74.7	815	2,046	4,350
Overall metabolite intake, mg/kg BW/day^1^						
Individual						
Isobutanol	0	0.3	0.6	3.4	8.0	15.4
2,3 Butanediol	10.5	0	0	216	609	1,318
Isobutyric acid	0	4.8	8.9	103	254	539
Total	10.5	5.1	9.5	322	871	1,872

^1^Mean metabolite intake over the 42-d feeding period, calculated from the weekly metabolite intakes (S5 Table).

### Broiler response variables: Growth performance

Overall (day 0 to 42) growth performance results are presented in Table 7; weekly results are presented in S6 Table. No known adverse health issues were observed throughout the conduct of the study. Early deaths occurred in all treatments and were consistent with expected mortality rates for the total number of animals assigned to study (S7 Table). The probable causes of death were varied but normal for chickens of this age and breed, were generally consistent across groups, and included, most commonly, airsacculitis, pericarditis and dehydration, often concurrently (S8 Table). There were no effects on mortality attributable to consumption of diets containing B10, B50 or B10 with supplemental metabolites at any targeted exposure level. Although mortality was numerically higher in the B10-10 group compared with other groups, several lines of evidence do not support conclusive attribution of this difference to an effect of the test diet. As expected with broiler chickens [86], the majority of total deaths (17/25) and deaths in the B10-10 group (6/8) occurred during the first two weeks of the study (S7 Table). There was no increase in mortality in the B10-10 group between the Starter (weeks 1–3) and Grower/Finisher (weeks 4–6) phases of the study, even with an approximate 80% increase in consumed metabolite dose (S5 Table) resulting from the increase in DDGS incorporation rate from 8% to 15% (Table 2) and concomitant scaling of the fermentation metabolite concentrations (Table 4). Noteworthy, the highest mortality for the B10-10 group was observed during week 2, which had the second lowest metabolite intake (1314.1 mg/kg BW/day), and no mortality was observed during week 5, when fermentation metabolite intake was highest for this group (2844.1 mg/kg BW/day). In the absence of unique observations in early decedents, adverse effects in the surviving broilers, or statistical significance when compared to either the eDDGS control or B10 groups, the weight of evidence does not support an effect of the test diet on mortality in the B10-10 group.

**Table 7:** Overall growth performance^1^ of broilers fed treatment diets from days 0 to 42.

Item	eDDGS	B10	B50	B10-2	B10-5	B10-10
Performance measure^2^^,^^3^						
Initial weight (g/bird)	39.5 (38.8 to 40.1)^6^	39.5 (38.9 to 40.2)	39.6 (38.9 to 40.3)	40.2 (39.6 to 40.9)	39.4 (38.7 to 40.1)	40.2 (39.6 to 40.9)
Final weight (g/bird)	2,610 (2,550 to 2,670)	2,580 (2,520 to 2,640)	2,570 (2,510 to 2,630)	2,590 (2,530 to 2,650)	2,570 (2,510 to 2,630)	2,500 (2,440 to 2,560)
Gain (g/bird)	2,570 (2,510 to 2,630)	2,540 (2,480 to 2,600)	2,530 (2,480 to 2,590)	2,550 (2,490 to 2,610)	2,530 (2,470 to 2,590)	2,460 (2,400 to 2,520)
Feed Intake (kg /pen)	43.3 (40.4 to 46.3)	42.0 (39.1 to 45.0)	41.7 (38.7 to 44.6)	43.1 (40.1 to 46.0)	42.2 (39.3 to 45.1)	38.1 (35.2 to 41.0)
Feed:gain (g/g)	1.79 (1.74 to 1.83)	1.75 (1.71 to 1.79)	1.74 (1.70 to 1.79)	1.78 (1.74 to 1.83)	1.78 (1.74 to 1.82)	1.77 (1.73 to 1.81)
Mortality^4^^,^^5^						
Initial birds, n	50	50	50	50	50	50
Final birds, n	47	46	47	47	46	42
Mortalities, n	3	4	3	3	4	8
Mortality, %	6.00	8.00	6.00	6.00	8.00	16.0

^1^Feed intake and mortality-corrected conversion least square means of 5 pens per treatment with 10 broilers per pen; initial weight least square means of 50 broilers per treatment, and final BW and gain least square means of 47, 46, 47, 47, 46, 42 broilers for eDDGS, B10, B50, B10-2, B10-5, and B10-10, respectively.

^2^Comparison with eDDGS control: treatment means did not differ, FDR-adjusted *P* > 0.05.

^3^Comparison with B10: treatment means did not differ, FDR-adjusted *P* > 0.05.

^4^Comparison with eDDGS control: treatment means did not differ, Fisher’s exact test *P* > 0.05.

^5^Comparison with B10: treatment means did not differ, Fisher’s exact test *P* > 0.05.

^6^Values in parentheses represent the CI of least squares means.

Body weight gains for all treatments were comparable to or slightly higher than those observed by other investigators [51, 87] when broilers were fed 16% or 18% DDGS for a 42-day period; mortality-corrected feed conversion or conversion converted to efficiency (gain, g:feed, kg, calculated as [1/g feed: g gain] x 1000]), however, were similar. There were no statistically significant differences in BW or weekly and overall BWG, feed consumption, or mortality-corrected feed:gain ratios when B10, B50, B10-2, B10-5 and B10-10 treatments were compared to eDDGS control, nor when B50, B10-2, B10-5 and B10-10 treatments were compared to B10 treatment. A trend toward lower BW, BWG, and feed consumption was observed for B10-10, however there was no effect on mortality-corrrected feed:gain ratios. Compared to eDDGS control, the mean BW and cumulative BWG were approximately 4% lower, feed intake was approximately 12% lower, and mortality-corrrected feed:gain ratios were approximately 3% lower. Compared to B10 treatment, B10-10 mean BW and cumulative BWG were approximately 3% lower, feed intake was approximately 9% lower, and mortality-corrrected feed:gain ratios were approximately 2% higher. Given that BWG was less impacted than ADFI, the magnitude of the observed differences, and in the absence of differences in other measures suggestive of treatment-related adverse effects or systemic toxicity, the slightly lower performance observed for the B10-10 group was therefore considered likely representative of a non-adverse palatability effect of diets containing high concentrations of isobutanol, 2,3-butanediol and isobutyric acid; however, the potential for approaching a limit of tolerability of these metabolites for poultry cannot be excluded. These results are similar to those reported for rats administered isobutanol orally at a dosage of 1000 mg/kg BW/day for 90 days [38]; however, there was no hypoactivity or other associated clinical signs of intoxication in poultry as were observed in rats.

### Broiler response variables: Hematology and clinical chemistry

There were no treatment-related effects on hematological or clinical chemistry parameters, and group means for most endpoints were comparable to the eDDGS control or B10 mean values. There were no statistical differences in any group when compared to the eDDGS control or B10 treatments for hematological or clinical chemistry endpoints (Tables 8 and 9). Some hematology (absolute and percent bands and eosinophils) and clinical chemistry (alanine aminotransferase, bilirubin, BUN, creatinine, BUN:creatinine, and albumin:globulin) endpoints could not be analyzed statistically due to the uniformity of the data. For hematology and clinical chemistry parameters with reference ranges, values were within the ranges established for avians by the analytical laboratory or similar to other published references [88–97]. Mean creatinine was higher in the B10-5 and B10-10 treatments with a resultant decrease in the BUN:creatinine ratios. However, there were no correlative effects on BUN or other clinical chemistry parameters, no histological correlates in the kidneys and skeletal muscle (S9 and S10 Tables), and mean values for all groups were within published reference ranges (0.1 to 0.4 mg/dL) [98]. Thus, the higher values were considered incidental and unrelated to consumption of the diets. Creatine phosphokinase could not be statistically analyzed as values above the limits of the standard curve were observed in some broilers across all treatments; these results were recorded as a uniform value of 150,000 U/L. Thus, the precision of the higher mean values in B10-5 and B10-10 compared to eDDGS could not be determined. However, there were no correlative effects on aspartate aminotransferase or other clinical chemistry measures, nor were there any histological correlates in the skeletal muscle (S10 Table). Thus, the higher values observed for those two treatments could not be definitively attributed to consumption of the test diets.

**Table 8:** Hematology results.

Item	eDDGS	B10	B50	B10-2	B10-5	B10-10
Sample n^1^	25	25	24	24	22	25
Assay^2^^,^^3^						
WBC estimate, 1000/μL	8.67 (6.89 to 11.2)^4^	8.90 (7.05 to 11.6)	9.06 (7.14 to 11.9)	8.29 (6.60 to 10.7)	8.63 (6.77 to 11.4)	7.70 (6.20 to 9.82)
Hematocrit, %	28.1 (26.6 to 29.6)	28.6 (27.1 to 30.1)	29.4 (27.8 to 31.0)	28.7 (27.0 to 30.4)	28.1 (26.5 to 29.7)	28.8 (27.2 to 30.3)
	% of total WBC					
Heterophils	46.9 (36.5 to 57.3)	50.5 (40.1 to 60.9)	38.6 (28.0 to 49.2)	44.1 (33.5 to 54.7)	49.6 (38.6 to 60.5)	51.4 (41.0 to 61.9)
Bands	0.00	2.52	0.00	0.00	0.00	0.00
Lymphocytes	39.2 (25.3 to 60.8)	24.3 (15.7 to 37.6)	48.5 (31.2 to 75.6)	38.2 (24.6 to 59.5)	34.1 (21.6 to 53.7)	30.3 (19.6 to 47.0)
Monocytes	2.58 (0.955 to 4.99)	4.40 (2.16 to 7.44)	1.29 (0.250 to 3.14)	3.01 (1.21 to 5.63)	3.68 (1.58 to 6.64)	5.34 (2.83 to 8.64)
Eosinophils	0.320	0.360	0.167	0.167	0.00	0.0400
Basophils	0.894 (0.177 to 2.16)	2.36 (1.02 to 4.24)	0.685 (0.0876 to 1.85)	2.27 (0.954 to 4.16)	0.458 (0.0151 to 1.51)	1.03 (0.241 to 2.37)
	Absolute /μL					
Heterophils	3,460 (2,390 to 5,020)	4,220 (2,890 to 6,160)	2,860 (1,960 to 4,170)	3,390 (2,330 to 4,950)	4,010 (2,700 to 5,940)	3,540 (2,440 to 5,140)
Bands	0.00	754	0.00	0.00	0.00	0.00
Lymphocytes	3,560 (2,080 to 6,080)	2,400 (1,400 to 4,110)	4,510 (2,620 to 7,750)	3,510 (2,040 to 6,040)	3,270 (1,870 to 5,710)	2,440 (1,430 to 4,180)
Monocytes	245 (88.4 to 480)	370 (168 to 649)	124 (23.0 to 305)	251 (90.3 to 491)	378 (166 to 678)	421 (204 to 717)
Eosinophils	45.6	35.4	26.9	11.9	0.00	3.00
Basophils	91.9 (12.1 to 247)	193 (60.5 to 400)	67.8 (4.23 to 208)	270 (105 to 510)	37.7 (0.0639 to 157)	81.7 (8.55 to 230)

^1^Hematocrit analysis, n = 17, 16, 15, 13, 15, 16, respectively for eDDGS, B10, B50, B10-2, B10-5, and B10-10; heterophils analysis, n = 23 for B10.

^2^Comparison with eDDGS control: treatment means did not differ, FDR-adjusted *P* > 0.05.

^3^Comparison with B10: treatment means did not differ, FDR-adjusted *P* > 0.05.

^4^Vaues in parentheses represent the CI of least squares means; CIs were not generated for endpoints not analyzed statistically due to the uniformity of the data.

**Table 9:** Serum chemistry results.

Item	eDDGS	B10	B50	B10-2	B10-5	B10-10
Sample n	25	25	24	24	24	25
Assay^1^^,^^2^						
AST^3^, U/L	273 (237 to 322)^4^	278 (241 to 328)	288 (248 to 344)	308 (263 to 373)	291 (250 to 348)	258 (226 to 301)
ALT^3^, U/L	5.32	5.00	5.00	5.71	5.42	5.16
ALP^3^, U/L^5^	6,530 (4,520 to 9,430)	4,130 (2,840 to 6,000)	3,120 (2,130 to 4,560)	2,880 (1,980 to 4,180)	3,220 (2,220 to 4,680)	3,340 (2,310 to 4,820)
GGTP^3^, U/L	17.2 (14.2 to 20.5)	16.5 (13.6 to 19.7)	16.3 (13.3 to 19.5)	13.6 (10.9 to 16.6)	17.3 (14.2 to 20.6)	14.6 (11.8 to 17.6)
Total bilirubin, mg/dL	0.104	0.104	0.117	0.100	0.104	0.104
Urea nitrogen, mg/dL	5.12	5.00	5.00	5.42	5.21	5.00
Creatinine, mg/dL	0.200	0.200	0.200	0.204	0.250	0.312
BUN:creatinine	25.6	25.0	25.0	26.8	22.0	17.0
Cholesterol, mg/dL	117 (105 to 128)	107 (95.9 to 119)	124 (112 to 136)	112 (101 to 124)	109 (97.6 to 121)	129 (117 to 140)
Triglycerides, mg/dL	36.4 (31.0 to 43.3)	30.5 (26.3 to 35.7)	33.7 (28.8 to 39.9)	31.8 (27.3 to 37.4)	32.4 (27.8 to 38.2)	33.3 (28.6 to 39.4)
Glucose, mg/dL	228 (217 to 239)	235 (223 to 246)	238 (226 to 249)	225 (214 to 237)	229 (218 to 241)	236 (225 to 248)
Total protein, g/dL	3.23 (3.08 to 3.38)	3.18 (3.03 to 3.33)	3.37 (3.22 to 3.52)	3.21 (3.06 to 3.36)	3.24 (3.09 to 3.39)	3.20 (3.06 to 3.35)
Albumin, g/dL	1.16 (1.10 to 1.22)	1.09 (1.03 to 1.16)	1.13 (1.07 to 1.19)	1.11 (1.04 to 1.17)	1.08 (1.01 to 1.14)	1.10 (1.03 to 1.16)
Globulin, g/dL	2.07 (1.96 to 2.18)	2.09 (1.98 to 2.20)	2.24 (2.12 to 2.35)	2.10 (1.98 to 2.21)	2.17 (2.05 to 2.28)	2.11 (2.00 to 2.22)
Albumin:globulin	0.752	0.528	0.504	0.546	0.700	0.520
Amylase, U/L	352 (308 to 406)	377 (329 to 438)	400 (346 to 467)	391 (339 to 456)	362 (315 to 420)	398 (345 to 463)
Lipase, U/L	10.8 (9.86 to 12.2)	9.92 (9.15 to 10.9)	10.5 (9.58 to 11.7)	9.90 (9.12 to 10.9)	10.7 (9.76 to 12.1)	11.5 (10.3 to 13.1)
CPK^3^, U/L	29,300	34,100	24,700	38,000	38,300	43,400
Phosphorus, mg/dL	6.55 (6.17 to 7.00)	6.57 (6.19 to 7.03)	6.36 (6.02 to 6.78)	6.37 (6.02 to 6.78)	6.55 (6.17 to 7.01)	6.40 (6.05 to 6.82)
Calcium, mg/dL	9.77 (9.56 to 9.96)	9.90 (9.70 to 10.1)	9.91 (9.70 to 10.1)	9.98 (9.78 to 10.2)	9.77 (9.56 to 9.97)	9.72 (9.51 to 9.92)
Magnesium, mEq/L	1.81 (1.73 to 1.89)	1.75 (1.67 to 1.83)	1.85 (1.76 to 1.93)	1.88 (1.79 to 1.96)	1.70 (1.61 to 1.78)	1.76 (1.68 to 1.84)
Sodium, mEq/L	145 (143 to 147)	145 (143 to 147)	145 (142 to 147)	145 (143 to 147)	146 (144 to 148)	146 (144 to 148)
Potassium, mEq/L	7.57 (6.21 to 8.93)	7.09 (5.73 to 8.45)	7.70 (6.34 to 9.06)	7.56 (6.20 to 8.93)	7.11 (5.75 to 8.47)	6.98 (5.62 to 8.34)
Sodium:Potassium	21.1 (16.5 to 24.3)	21.9 (17.7 to 24.9)	19.9 (14.4 to 23.4)	20.3 (15.1 to 23.7)	21.5 (17.1 to 24.6)	22.4 (18.4 to 25.2)
Chloride, mEq/L	111 (109 to 112)	112 (110 to 113)	111 (110 to 113)	112 (111 to 114)	113 (111 to 115)	112 (111 to 114)

^1^Comparison with eDDGS control: treatment means did not differ, FDR-adjusted *P* > 0.05.

^2^Comparison with B10: treatment means did not differ, FDR-adjusted *P* > 0.05.

^3^Aspartate aminotransferase (AST); alanine aminotransferase (ALT); alkaline phosphatase (ALP); gamma glutamyl-transpeptidase (GGTP); creatine phosphokinase (CPK).

^4^Values in parentheses represent the CI of least squares means; CIs were not generated for endpoints not analyzed statistically due to the uniformity of the data.

^5^n = 24 and n = 23 for B10 and B50, respectively.

### Broiler response variables: Anatomic pathology

There were no gross findings in broilers at scheduled sacrifice. Organ weights (absolute and relative to d 42 BW) are presented in Table 10. There were no statistical differences in any group when compared to the eDDGS control or B10 treatments for all measures except the absolute weight for the bursa of Fabricius. Bursal weights were statistically lower in broilers fed B10-10 compared to those fed B10. However, there were no histological correlates to the weight differences (S11 Table) and the bursal weights were not statistically different compared to broilers fed eDDGS. Thus, these weight differences were considered incidental findings associated with normal physiological variation and could not be definitively attributed to treatment diet consumption. As described previously, irritation of the upper respiratory tract has been observed in inhalation toxicity studies of isobutyric acid in rats [39, 40], suggesting a potential risk for similar effects in poultry inhaling these volatile metabolites. However, there was no histological evidence of increased pulmonary irritation in the groups fed diets containing supplemental isobutyric acid (S12 Table). All microscopic findings observed in this study were typical of background lesions in chickens of this age and breed and occurred across all treatments.

**Table 10:** Absolute and relative (to final body weight) organ weight.

Item	eDDGS	B10	B50	B10-2	B10-5	B10-10
Sample n	25	25	24	24	24	25
	Weight (g)^1^					
Brain	2.33 (2.10 to 2.56)^4^	2.61 (2.38 to 2.84)	2.40 (2.17 to 2.63)	2.36 (2.13 to 2.59)	2.41 (2.18 to 2.64)	2.39 (2.16 to 2.62)
Liver	62.1 (57.6 to 66.6)	64.0 (59.5 to 68.5)	58.1 (53.5 to 62.7)	61.5 (56.9 to 66.1)	63.1 (58.5 to 67.7)	57.0 (52.5 to 61.5)
Kidney	15.0 (13.3 to 16.8)	14.9 (13.2 to 16.6)	12.7 (11.0 to 14.4)	15.0 (13.3 to 16.8)	16.4 (14.7 to 18.1)	13.3 (11.6 to 15.0)
Heart	15.3 (13.5 to 17.1)	13.7 (11.8 to 15.5)	13.0 (11.2 to 14.9)	15.4 (13.6 to 17.3)	15.3 (13.4 to 17.1)	15.1 (13.2 to 16.9)
Bursa of Fabricius	3.25 (2.88 to 3.66)	3.64 (3.23 to 4.11)	3.50 (3.10 to 3.95)	3.66 (3.24 to 4.14)	3.13 (2.77 to 3.54)	2.57^3^ (2.28 to 2.90)
Lung	19.6 (18.0 to 21.2)	18.7 (17.0 to 20.3)	20.2 (18.6 to 21.8)	19.1 (17.5 to 20.7)	19.3 (17.7 to 20.9)	17.2 (15.6 to 18.8)
Testes	0.671 (0.592 to 0.761)	0.650 (0.573 to 0.737)	0.568 (0.499 to 0.646)	0.764 (0.671 to 0.868)	0.770 (0.677 to 0.876)	0.736 (0.649 to 0.835)
Crop	6.41 (5.71 to 7.23)	5.26 (4.74 to 5.87)	5.95 (5.32 to 6.70)	5.56 (4.99 to 6.23)	5.40 (4.85 to 6.04)	5.32 (4.79 to 5.94)
	% of 42-d BW^1^^,^^2^					
Brain	0.0896 (0.0802 to 0.0991)	0.101 (0.0915 to 0.110)	0.0939 (0.0844 to 0.103)	0.0932 (0.0836 to 0.103)	0.0940 (0.0844 to 0.104)	0.0965 (0.0871 to 0.106)
Liver	2.40 (2.23 to 2.56)	2.47 (2.31 to 2.64)	2.26 (2.09 to 2.43)	2.42 (2.25 to 2.59)	2.46 (2.29 to 2.63)	2.32 (2.16 to 2.49)
Kidney	0.578 (0.516 to 0.640)	0.577 (0.516 to 0.639)	0.495 (0.432 to 0.557)	0.593 (0.531 to 0.656)	0.638 (0.576 to 0.701)	0.538 (0.476 to 0.600)
Heart	0.591 (0.520 to 0.661)	0.529 (0.458 to 0.599)	0.509 (0.438 to 0.580)	0.612 (0.541 to 0.683)	0.592 (0.521 to 0.663)	0.611 (0.541 to 0.682)
Bursa of Fabricius	0.125 (0.111 to 0.141)	0.140 (0.124 to 0.158)	0.136 (0.120 to 0.154)	0.144 (0.128 to 0.163)	0.121 (0.107 to 0.137)	0.104 (0.0919 to 0.117)
Lung	0.761 (0.693 to 0.829)	0.722 (0.654 to 0.790)	0.789 (0.720 to 0.858)	0.758 (0.689 to 0.827)	0.748 (0.679 to 0.817)	0.699 (0.632 to 0.767)
Testes	0.0259 (0.0229 to 0.0292)	0.0251 (0.0222 to 0.0283)	0.0222 (0.0196 to 0.0251)	0.0301 (0.0266 to 0.0341)	0.0297 (0.0263 to 0.0337)	0.0297 (0.0263 to 0.0335)
Crop	0.246 (0.220 to 0.278)	0.203 (0.183 to 0.226)	0.230 (0.206 to 0.259)	0.219 (0.196 to 0.245)	0.208 (0.187 to 0.233)	0.214 (0.192 to 0.239)

^1^Comparison with eDDGS control: treatment means did not differ, FDR-adjusted *P* > 0.05.

^2^Comparison with B10: treatment means did not differ, FDR-adjusted *P* > 0.05.

^3^Comparison with B10: FDR-adjusted *P* < 0.05.

^4^Values in parentheses represent CI of least squares means.

## Conclusions

The results of this feeding trial indicate that broiler consumption of phase diets containing simulated DDGS with 10% or 50% BMY generated from a novel isobutanol production process and fed at 8% or 15% of the diet, and containing isobutanol, 2,3-butanediol and isobutyric acid at 1, 3, and 6 times the anticipated specification limits was well-tolerated. Reduced live performance in broilers from the B10-10 group was likely attributable to a non-adverse palatability effect of diets containing high metabolite concentrations; however, these subtle differences may suggest that an upper limit of tolerability is being approached. The weight of evidence from this evaluation suggests that consumption by poultry of diets containing butanol DDGS, heat-inactivated BMY, and associated fermentation metabolites presents a reasonable certainty of no harm under the anticipated conditions of use. Additionally, given the absence of published feeding trials using genetically engineered yeast, the protocol presented herein serves as a model for evaluating the safety of genetically engineered yeast in poultry.

## Supporting information

S1 Table Mycotoxin analyses (as-is basis) of eDDGS, B10 and B50 DDGS sources. (DOCX)

S2 Table Nutrient composition analyses (as-fed basis) of eDDGS, B10, and B50 starter phase diets at the start (day 0) and end (day 21) of the feeding period. (DOCX)

S3 Table Nutrient composition analyses (as-fed basis) of eDDGS, B10, and B50 grower phase diets at the start (day 22) and end (day 35) of the feeding period. (DOCX)

S4 Table Nutrient composition analyses (as-fed basis) of eDDGS, B10, and B50 finisher phase diets at the start (day 36) and end (day 42) of the feeding period. (DOCX)

S5 Table Weekly intakes of isobutanol, 2,3-butanediol, and isobutyric acid in treatment phase diets. (DOCX)

S6 Table Weekly growth performance measures^1^ of broilers fed treatment diets from day 0 to day 42. (DOCX)

S7 Table Weekly mortality. (DOCX)

S8 Table Macroscopic observations for early-decedent broilers. (DOCX)

S9 Table Incidence and severity of histologic observations in the kidneys. (DOCX)

S10 Table Incidence and severity of histologic observations in the skeletal muscle. (DOCX)

S11 Table Incidence and severity of histologic observations in the bursa of Fabricius. (DOCX)

S12 Table Incidence and severity of histologic observations in the lungs. (DOCX)
