# Supplementary material for: Nutrient composition and safety evaluation of simulated isobutanol distillers dried grains with solubles and associated fermentation metabolites when fed to male Ross 708 broiler chickens (Gallus domesticus)
Source: PLoS One. 2019 Jul 8;14(7):e0219016. doi: 10.1371/journal.pone.0219016 (PMC6613701; doi:10.1371/journal.pone.0219016)
Supplement: S1 Table — (DOCX) [file pone.0219016.s001.docx]

S1 Table. Mycotoxin analyses (as-is basis) of eDDGS, B10 and B50 DDGS sources.

|  | Source | | |
| --- | --- | --- | --- |
| Mycotoxin (ppb) | eDDGS | B10 | B50 |
| Aflatoxin B1 | ND^1,2^ | ND | ND |
| Aflatoxin B2 | ND | ND | ND |
| Aflatoxin G1 | ND | ND | ND |
| Aflatoxin G2 | ND | ND | ND |
| Ochratoxin A | ND | ND | ND |
| Zearalenone | 50.9 | 262 | 153 |
| T2-Toxin | ND | ND | ND |
| Deoxynivalenol (DON) | 668 | ND | ND |
| 3/15-Acetyl-DON | ND | ND | ND |
| Fumonisin B1 | 299 | 775 | 380 |
| Fumonisin B2 | 83.6 | 433 | 208 |
| Fumonisin B3 | 29.4 | 127 | 61.8 |

^1^ND – not detected above the limit of quantification or limit of detection (LOQ/LOD). ^2^LOQ and LOD values for DON and 3/15-Acetyl-DON are 160 ppb and 50 ppb, respectively; LOQ and LOD values for all other analytes are 4 ppb and 1 ppb, respectively.
