# Supplementary material for: Nutrient composition and safety evaluation of simulated isobutanol distillers dried grains with solubles and associated fermentation metabolites when fed to male Ross 708 broiler chickens (Gallus domesticus)
Source: PLoS One. 2019 Jul 8;14(7):e0219016. doi: 10.1371/journal.pone.0219016 (PMC6613701; doi:10.1371/journal.pone.0219016)
Supplement: S2 Table — (DOCX) [file pone.0219016.s002.docx]

S2 Table. Nutrient composition analyses (as-fed basis) of eDDGS, B10, and B50 starter phase diets at the start (day 0) and end (day 21) of the feeding period.

| Treatment | eDDGS | |  | B10 | |  | B50 | |
| --- | --- | --- | --- | --- | --- | --- | --- | --- |
| Sample time | Start | End |  | Start | End |  | Start | End |
| Item |  |  |  |  |  |  |  |  |
| Proximates, energy, and minerals (% except as noted) | | | | | | | | |
| Moisture | 13.1 | 11.8 |  | 13.0 | 12.0 |  | 13.3 | 12.0 |
| CP | 20.7 | 20.4 |  | 20.0 | 20.2 |  | 20.3 | 20.0 |
| Crude fat | 8.60 | 8.06 |  | 6.86 | 8.40 |  | 7.05 | 7.28 |
| Gross energy, kcal/kg | 4,060 | 4,140 |  | 4,060 | 4,160 |  | 3,980 | 4,020 |
| Crude fiber | 2.75 | 3.04 |  | 3.04 | 3.12 |  | 2.76 | 2.51 |
| Ash | 5.02 | 4.90 |  | 4.75 | 4.46 |  | 4.45 | 4.83 |
| Calcium | 0.972 | 0.889 |  | 0.982 | 0.915 |  | 0.913 | 1.12 |
| Phosphorus | 0.799 | 0.768 |  | 0.755 | 0.746 |  | 0.829 | 0.826 |
|  | | | | | | | | |
| Essential amino acid, % | | | | | | | | |
| Arg | 1.24 | 1.15 |  | 1.22 | 1.23 |  | 1.16 | 1.17 |
| His | 0.575 | 0.445 |  | 0.543 | 0.532 |  | 0.508 | 0.519 |
| Ile | 0.910 | 0.722 |  | 0.881 | 0.924 |  | 0.889 | 0.896 |
| Leu | 1.81 | 1.49 |  | 1.77 | 1.83 |  | 1.74 | 1.73 |
| Lys | 1.23 | 1.20 |  | 1.17 | 1.21 |  | 1.23 | 1.24 |
| Met | 0.614 | 0.668 |  | 0.637 | 0.604 |  | 0.681 | 0.775 |
| Met + Cys | 0.881 | 0.971 |  | 0.942 | 0.869 |  | 0.998 | 1.03 |
| Phe | 1.04 | 0.846 |  | 1.04 | 1.04 |  | 0.972 | 0.981 |
| Thr | 0.806 | 0.790 |  | 0.791 | 0.810 |  | 0.792 | 0.792 |
| Trp | 0.222 | 0.202 |  | 0.210 | 0.205 |  | 0.191 | 0.209 |
| Val | 0.984 | 0.785 |  | 0.959 | 0.990 |  | 0.974 | 0.971 |
|  | | | | | | | | |
| Non-essential amino acid, % | | | | | | | | |
| Ala | 1.04 | 0.865 |  | 1.02 | 1.05 |  | 1.05 | 1.04 |
| Asp | 2.10 | 1.70 |  | 2.00 | 2.10 |  | 2.07 | 2.11 |
| Cys | 0.267 | 0.303 |  | 0.305 | 0.265 |  | 0.317 | 0.258 |
| Glu | 3.75 | 2.83 |  | 3.57 | 3.74 |  | 3.56 | 3.61 |
| Gly | 0.865 | 0.711 |  | 0.850 | 0.856 |  | 0.833 | 0.831 |
| Pro | 1.24 | 0.992 |  | 1.20 | 1.24 |  | 1.16 | 1.16 |
| Ser | 1.03 | 0.764 |  | 1.01 | 1.03 |  | 0.980 | 0.978 |
| Tyr | 0.555 | 0.452 |  | 0.557 | 0.567 |  | 0.544 | 0.524 |
