# Supplementary material for: Nutrient composition and safety evaluation of simulated isobutanol distillers dried grains with solubles and associated fermentation metabolites when fed to male Ross 708 broiler chickens (Gallus domesticus)
Source: PLoS One. 2019 Jul 8;14(7):e0219016. doi: 10.1371/journal.pone.0219016 (PMC6613701; doi:10.1371/journal.pone.0219016)
Supplement: S3 Table — (DOCX) [file pone.0219016.s003.docx]

S3 Table. Nutrient composition analyses (as-fed basis) of eDDGS, B10, and B50 grower phase diets at the start (day 22) and end (day 35) of the feeding period.

| Treatment | eDDGS | |  | B10 | |  | B50 | |
| --- | --- | --- | --- | --- | --- | --- | --- | --- |
| Sample time | Start | End |  | Start | End |  | Start | End |
| Item |  |  |  |  |  |  |  |  |
| Proximates, energy, and minerals (% except as noted) | | | | | | | | |
| Moisture | 14.1 | 12.3 |  | 13.7 | 12.7 |  | 14.3 | 12.8 |
| CP | 19.2 | 19.5 |  | 18.7 | 19.0 |  | 19.2 | 18.6 |
| Crude fat | 10.0 | 9.32 |  | 10.3 | 9.71 |  | 7.55 | 7.87 |
| GE, kcal/kg | 4,030 | 4,220 |  | 4,160 | 4,180 |  | 4,080 | 4,100 |
| Crude fiber | 2.96 | 2.86 |  | 3.66 | 3.56 |  | 2.78 | 2.55 |
| Ash | 4.51 | 4.76 |  | 4.05 | 4.26 |  | 4.04 | 4.45 |
| Calcium | 0.795 | 0.890 |  | 0.836 | 0.872 |  | 0.805 | 1.03 |
| Phosphorus | 0.764 | 0.825 |  | 0.715 | 0.741 |  | 0.748 | 0.802 |
|  | | | | | | | | |
| Essential amino acid, % | | | | | | | | |
| Arg | 1.07 | 1.10 |  | 1.06 | 1.08 |  | 1.11 | 0.990 |
| His | 0.495 | 0.502 |  | 0.470 | 0.499 |  | 0.523 | 0.450 |
| Ile | 0.775 | 0.811 |  | 0.798 | 0.791 |  | 0.874 | 0.788 |
| Leu | 1.67 | 1.71 |  | 1.72 | 1.74 |  | 1.77 | 1.61 |
| Lys | 1.23 | 1.16 |  | 1.15 | 1.11 |  | 1.19 | 1.13 |
| Met | 0.574 | 0.583 |  | 0.557 | 0.560 |  | 0.531 | 0.589 |
| Met + Cys | 0.869 | 0.807 |  | 0.858 | 0.803 |  | 0.760 | 0.803 |
| Phe | 0.909 | 0.950 |  | 0.922 | 0.949 |  | 1.01 | 0.867 |
| Thr | 0.712 | 0.739 |  | 0.732 | 0.730 |  | 0.815 | 0.721 |
| Trp | 0.178 | 0.180 |  | 0.186 | 0.161 |  | 0.195 | 0.162 |
| Val | 0.858 | 0.900 |  | 0.889 | 0.878 |  | 0.977 | 0.879 |
|  | | | | | | | | |
| Non-essential amino acid, % | | | | | | | | |
| Ala | 0.977 | 0.994 |  | 1.01 | 1.02 |  | 1.09 | 1.00 |
| Asp | 1.78 | 1.82 |  | 1.82 | 1.83 |  | 1.89 | 1.80 |
| Cys | 0.295 | 0.224 |  | 0.301 | 0.243 |  | 0.229 | 0.214 |
| Glu | 3.28 | 3.38 |  | 3.37 | 3.37 |  | 3.33 | 3.13 |
| Gly | 0.773 | 0.800 |  | 0.771 | 0.777 |  | 0.844 | 0.750 |
| Pro | 1.14 | 1.18 |  | 1.17 | 1.17 |  | 1.18 | 1.07 |
| Ser | 0.910 | 0.945 |  | 0.925 | 0.931 |  | 0.962 | 0.879 |
| Tyr | 0.493 | 0.524 |  | 0.508 | 0.509 |  | 0.569 | 0.471 |
