# Supplementary material for: Nutrient composition and safety evaluation of simulated isobutanol distillers dried grains with solubles and associated fermentation metabolites when fed to male Ross 708 broiler chickens (Gallus domesticus)
Source: PLoS One. 2019 Jul 8;14(7):e0219016. doi: 10.1371/journal.pone.0219016 (PMC6613701; doi:10.1371/journal.pone.0219016)
Supplement: S4 Table — (DOCX) [file pone.0219016.s004.docx]

S4 Table. Nutrient composition analyses (as-fed basis) of eDDGS, B10, and B50 finisher phase diets at the start (day 36) and end (day 42) of the feeding period.

| Treatment | eDDGS | |  | B10 | |  | B50 | |
| --- | --- | --- | --- | --- | --- | --- | --- | --- |
| Sample time | Start | End |  | Start | End |  | Start | End |
| Item |  |  |  |  |  |  |  |  |
| Proximates, energy, and minerals (% except as noted) | | | | | | | | |
| Moisture | 14.3 | 13.7 |  | 14.4 | 13.6 |  | 14.5 | 12.7 |
| CP | 18.0 | 17.6 |  | 17.5 | 18.2 |  | 18.7 | 18.6 |
| Crude fat | 10.9 | 11.3 |  | 10.8 | 12.9 |  | 7.32 | 8.68 |
| GE, kcal/kg | 4,170 | 4,260 |  | 4,210 | 4,220 |  | 4,080 | 4,120 |
| Crude fiber | 2.93 | 2.95 |  | 3.56 | 3.48 |  | 2.78 | 2.92 |
| Ash | 4.23 | 4.14 |  | 3.63 | 3.69 |  | 3.79 | 3.57 |
| Calcium | 0.737 | 0.769 |  | 0.729 | 0.781 |  | 0.690 | 0.670 |
| Phosphorus | 0.716 | 0.690 |  | 0.635 | 0.674 |  | 0.668 | 0.675 |
|  | | | | | | | | |
| Essential amino acid, % | | | | | | | | |
| Arg | 0.988 | 1.03 |  | 1.06 | 0.936 |  | 1.00 | 0.941 |
| His | 0.482 | 0.481 |  | 0.476 | 0.429 |  | 0.465 | 0.458 |
| Ile | 0.732 | 0.773 |  | 0.777 | 0.724 |  | 0.804 | 0.792 |
| Leu | 1.61 | 1.66 |  | 1.69 | 1.58 |  | 1.65 | 1.63 |
| Lys | 1.04 | 1.06 |  | 1.01 | 1.08 |  | 1.14 | 1.14 |
| Met | 0.543 | 0.513 |  | 0.560 | 0.518 |  | 0.489 | 0.527 |
| Met + Cys | 0.833 | 0.760 |  | 0.837 | 0.763 |  | 0.705 | 0.750 |
| Phe | 0.861 | 0.910 |  | 0.961 | 0.839 |  | 0.909 | 0.857 |
| Thr | 0.682 | 0.705 |  | 0.729 | 0.662 |  | 0.750 | 0.732 |
| Trp | 0.178 | 0.165 |  | 0.178 | 0.165 |  | 0.167 | 0.159 |
| Val | 0.817 | 0.866 |  | 0.871 | 0.806 |  | 0.908 | 0.896 |
|  | | | | | | | | |
| Non-essential amino acid, % | | | | | | | | |
| Ala | 0.938 | 0.952 |  | 0.970 | 0.922 |  | 1.04 | 1.06 |
| Asp | 1.65 | 1.66 |  | 1.61 | 1.58 |  | 1.76 | 1.75 |
| Cys | 0.290 | 0.247 |  | 0.277 | 0.245 |  | 0.216 | 0.223 |
| Glu | 3.11 | 3.17 |  | 3.09 | 3.00 |  | 3.08 | 3.06 |
| Gly | 0.730 | 0.761 |  | 0.775 | 0.691 |  | 0.783 | 0.749 |
| Pro | 1.11 | 1.14 |  | 1.16 | 1.07 |  | 1.10 | 1.09 |
| Ser | 0.867 | 0.904 |  | 0.918 | 0.829 |  | 0.881 | 0.854 |
| Tyr | 0.464 | 0.494 |  | 0.541 | 0.479 |  | 0.515 | 0.486 |
