# Supplementary material for: Nutrient composition and safety evaluation of simulated isobutanol distillers dried grains with solubles and associated fermentation metabolites when fed to male Ross 708 broiler chickens (Gallus domesticus)
Source: PLoS One. 2019 Jul 8;14(7):e0219016. doi: 10.1371/journal.pone.0219016 (PMC6613701; doi:10.1371/journal.pone.0219016)
Supplement: S5 Table — (DOCX) [file pone.0219016.s005.docx]

S5 Table. Weekly intakes of isobutanol, 2,3-butanediol, and isobutyric acid in treatment phase diets.

| Metabolite | eDDGS | B10 | B50 | B10-2 | B10-5 | B10-10 |
| --- | --- | --- | --- | --- | --- | --- |
| Isobutanol, mg/kg BW/day | | | | | | |
| d 0 to 7 | 0 | 0.2 | 0.6 | 4.4 | 8.9 | 15.0 |
| d 7 to 14 | 0 | 0.2 | 0.4 | 3.2 | 6.4 | 10.2 |
| d 14 to 21 | 0 | 0.2 | 0.4 | 2.6 | 5.2 | 7.9 |
| d 21 to 28 | 0 | 0.4 | 0.8 | 3.6 | 9.6 | 17.7 |
| d 28 to 35 | 0 | 0.4 | 0.8 | 3.6 | 8.8 | 21.1 |
| d 35 to 42 | 0 | 0.2 | 0.5 | 3.0 | 8.9 | 20.3 |
|  |  |  |  |  |  |  |
| 2,3-Butanediol, mg/kg BW/day | | | | | | |
| d 0 to 7 | 0 | 0 | 0 | 202 | 579 | 1,305 |
| d 7 to 14 | 0 | 0 | 0 | 159 | 451 | 959 |
| d 14 to 21 | 0 | 0 | 0 | 147 | 401 | 797 |
| d 21 to 28 | 25.4 | 0 | 0 | 267 | 743 | 1,536 |
| d 28 to 35 | 28.6 | 0 | 0 | 315 | 830 | 1,964 |
| d 35 to 42 | 8.9 | 0 | 0 | 208 | 647 | 1,347 |
|  |  |  |  |  |  |  |
| Isobutyric acid, mg/kg BW/day | | | | | | |
| d 0 to 7 | 0 | 4.0 | 5.7 | 110 | 234 | 479 |
| d 7 to 14 | 0 | 3.0 | 4.3 | 78.6 | 178 | 345 |
| d 14 to 21 | 0 | 2.7 | 3.7 | 69.0 | 158 | 291 |
| d 21 to 28 | 0 | 6.2 | 14.2 | 114 | 320 | 655 |
| d 28 to 35 | 0 | 7.2 | 14.5 | 133 | 352 | 859 |
| d 35 to 42 | 0 | 5.8 | 10.9 | 112 | 283 | 606 |
