# Supplementary material for: Nutrient composition and safety evaluation of simulated isobutanol distillers dried grains with solubles and associated fermentation metabolites when fed to male Ross 708 broiler chickens (Gallus domesticus)
Source: PLoS One. 2019 Jul 8;14(7):e0219016. doi: 10.1371/journal.pone.0219016 (PMC6613701; doi:10.1371/journal.pone.0219016)
Supplement: S6 Table — (DOCX) [file pone.0219016.s006.docx]

S6 Table. Weekly growth performance measures^1^ of broilers fed treatment diets from day 0 to day 42.

| Item | eDDGS | B10 | B50 | B10-2 | B10-5 | B10-10 |
| --- | --- | --- | --- | --- | --- | --- |
| BW (g) | | | | | | |
| day 0 | 39.5  (38.8 to 40.1)^2^ | 39.5  (38.9 to 40.2) | 39.6  (38.9 to 40.3) | 40.2  (39.6 to 40.9) | 39.4  (38.7 to 40.1) | 40.2  (39.6 to 40.9) |
| day 7 | 154  (152 to 157) | 154  (151 to 156) | 153  (151 to 155) | 154  (151 to 156) | 152  (149 to 154) | 153  (150 to 155) |
| day 14 | 387  (379 to 395) | 388  (380 to 396) | 384  (376 to 392) | 385  (377 to 393) | 382  (374 to 390) | 379  (371 to 388) |
| day 21 | 747  (731 to 762) | 742  (727 to 758) | 739  (724 to 755) | 743  (728 to 759) | 737  (722 to 753) | 726  (710 to 742) |
| day 28 | 1,230  (1,210 to 1,260) | 1,220  (1,190 to 1,250) | 1,210  (1,190 to 1,240) | 1,230  (1,200 to 1,250) | 1,220  (1,200 to 1,250) | 1,190  (1,160 to 1,220) |
| day 35 | 1,910  (1,870 to 1,950) | 1,900  (1,860 to 1,940) | 1,880  (1,840 to 1,930) | 1,900  (1,860 to 1,940) | 1,900  (1,850 to 1,940) | 1,850  (1,810 to 1,900) |
| day 42 | 2,610  (2,550 to 2,670) | 2,580  (2,520 to 2,640) | 2,570  (2,510 to 2,630) | 2,590  (2,530 to 2,650) | 2,570  (2,510 to 2,630) | 2,500  (2,440 to 2,560) |
|  | | | | | | |
| Gain (g) | | | | | | |
| day 0 to 7 | 115  (113 to 116) | 114  (112 to 116) | 113  (112 to 115) | 113  (112 to 115) | 112  (111 to 114) | 112  (110 to 114) |
| day 7 to 14 | 233  (227 to 239) | 234  (228 to 239) | 231  (225 to 237) | 231  (226 to 237) | 231  (225 to 237) | 227  (221 to 233) |
| day 14 to 21 | 358  (348 to 369) | 355  (344 to 365) | 355  (344 to 366) | 359  (349 to 370) | 355  (344 to 366) | 347  (335 to 358) |
| day 21 to 28 | 487  (475 to 499) | 480  (467 to 492) | 475  (463 to 488) | 483  (470 to 495) | 484  (472 to 497) | 467  (454 to 480) |
| day 28 to 35 | 677  (660 to 694) | 676  (660 to 693) | 669  (652 to 686) | 674  (657 to 691) | 673  (657 to 690) | 661  (643 to 679) |
| day 35 to 42 | 696  (673 to 719) | 680  (657 to 703) | 690  (667 to 712) | 687  (665 to 710) | 673  (651 to 696) | 639  (615 to 662) |
|  | | | | | | |
| Feed intake (kg) | | | | | | |
| day 0 to 7 | 1.31  (1.26 to 1.36) | 1.33  (1.28 to 1.39) | 1.33  (1.28 to 1.38) | 1.32  (1.27 to 1.38) | 1.26  (1.21 to 1.31) | 1.27  (1.22 to 1.32) |
| day 7 to 14 | 2.73  (2.54 to 2.91) | 2.72  (2.53 to 2.91) | 2.64  (2.45 to 2.83) | 2.72  (2.53 to 2.90) | 2.62  (2.43 to 2.80) | 2.46  (2.28 to 2.65) |
| day 14 to 21 | 5.02  (4.69 to 5.35) | 5.01  (4.69 to 5.34) | 4.74  (4.41 to 5.07) | 5.02  (4.69 to 5.35) | 5.02  (4.69 to 5.35) | 4.53  (4.20 to 4.86) |
| day 21 to 28 | 7.07  (6.33 to 7.80) | 7.10  (6.37 to 7.84) | 7.52  (6.79 to 8.26) | 7.30  (6.57 to 8.04) | 7.48  (6.75 to 8.22) | 6.63  (5.90 to 7.36) |
| day 28 to 35 | 12.6  (11.6 to 13.6) | 13.0  (12.0 to 14.0) | 12.2  (11.2 to 13.2) | 12.2  (11.2 to 13.2) | 12.0  (11.0 to 13.0) | 11.2  (10.2 to 12.2) |
| day 35 to 42 | 14.6  (13.0 to 16.1) | 12.9  (11.4 to 14.4) | 13.3  (11.8 to 14.8) | 14.5  (13.0 to 16.0) | 13.8  (12.3 to 15.3) | 12.0  (10.5 to 13.6) |
|  | | | | | | |
| Feed:Gain (g/g) | | | | | | |
| day 0 to 7 | 1.17  (1.14 to 1.19) | 1.17  (1.14 to 1.20) | 1.17  (1.15 to 1.20) | 1.17  (1.14 to 1.20) | 1.17  (1.14 to 1.19) | 1.18  (1.15 to 1.21) |
| day 7 to 14 | 1.22  (1.16 to 1.27) | 1.21  (1.16 to 1.26) | 1.22  (1.16 to 1.27) | 1.20  (1.14 to 1.25) | 1.21  (1.15 to 1.26) | 1.24  (1.18 to 1.29) |
| day 14 to 21 | 1.50  (1.43 to 1.56) | 1.47  (1.41 to 1.54) | 1.42  (1.36 to 1.48) | 1.49  (1.42 to 1.55) | 1.51  (1.44 to 1.57) | 1.48  (1.42 to 1.55) |
| day 21 to 28 | 1.54  (1.44 to 1.65) | 1.58  (1.47 to 1.68) | 1.68  (1.58 to 1.78) | 1.61  (1.51 to 1.71) | 1.65  (1.54 to 1.75) | 1.65  (1.54 to 1.75) |
| day 28 to 35 | 2.00  (1.88 to 2.12) | 2.05  (1.93 to 2.16) | 1.93  (1.82 to 2.05) | 1.93  (1.81 to 2.04) | 1.90  (1.79 to 2.02) | 1.96  (1.85 to 2.08) |
| day 35 to 42 | 2.21  (2.01 to 2.42) | 2.06  (1.86 to 2.27) | 2.05  (1.85 to 2.26) | 2.26  (2.05 to 2.47) | 2.23  (2.03 to 2.44) | 2.25  (2.04 to 2.46) |

^1^Feed intake and efficiency least square means represent 5 pens per treatment with 10 broilers per pen; initial weight least square means represent 50 broilers per treatment, and final BW and gain least square means represent 47, 46, 47, 47, 46, 42 broilers for eDDGS, B10, B50, B10-2, B10-5, and B10-10, respectively. ^2^Values in parentheses represent CI of least squares means.
