# Supplementary material for: Nutrient composition and safety evaluation of simulated isobutanol distillers dried grains with solubles and associated fermentation metabolites when fed to male Ross 708 broiler chickens (Gallus domesticus)
Source: PLoS One. 2019 Jul 8;14(7):e0219016. doi: 10.1371/journal.pone.0219016 (PMC6613701; doi:10.1371/journal.pone.0219016)
Supplement: S7 Table — (DOCX) [file pone.0219016.s007.docx]

S7 Table. Weekly mortality.

| Mortality, # | eDDGS | B10 | B50 | B10-2 | B10-5 | B10-10 |
| --- | --- | --- | --- | --- | --- | --- |
| day 0 to 7 | 1 (49)^1^ | 0 (50) | 0 (50) | 0 (50) | 2 (48) | 2 (48) |
| day 7 to 14 | 1 (48) | 2 (48) | 3 (47) | 1 (49) | 1 (47) | 4 (44) |
| day 14 to 21 | 1 (47) | 0 (48) | 0 (47) | 2 (47) | 0 (47) | 0 (44) |
| day 21 to 28 | 0 (47) | 1 (47) | 0 (47) | 0 (47) | 0 (47) | 1 (43) |
| day 28 to 35 | 0 (47) | 0 (47) | 0 (47) | 0 (47) | 0 (47) | 0 (43) |
| day 35 to 42 | 0 (47) | 1 (46) | 0 (47) | 0 (47) | 1 (46) | 1 (42) |

^1^Values in parentheses represent the number of surviving birds at the end of each week.
