# Supplementary material for: Nutrient composition and safety evaluation of simulated isobutanol distillers dried grains with solubles and associated fermentation metabolites when fed to male Ross 708 broiler chickens (Gallus domesticus)
Source: PLoS One. 2019 Jul 8;14(7):e0219016. doi: 10.1371/journal.pone.0219016 (PMC6613701; doi:10.1371/journal.pone.0219016)
Supplement: S8 Table — (DOCX) [file pone.0219016.s008.docx]

S8 Table. Macroscopic observations for early-decedent broilers.

| Treatment | Week of Death | Dead Body Weight (g) | Mortality Observations |
| --- | --- | --- | --- |
| eDDGS | 1 | 71.7 | litter in crop/gizzard, omphalitis, pericarditis, perihepatitis |
|  | 2 | 177.6 | airsacculitis, dehydration, pericarditis, perihepatitis |
|  | 3 | 401.5 | airsacculitis, dehydration, litter in crop/gizzard, pericarditis, perihepatitis |
| B10 | 2 | 150.2 | gizzard erosion, nonspecific enteritis, omphalitis, pericarditis |
|  | 2 | 173.7 | airsacculitis, gizzard erosion, pericarditis, perihepatitis |
|  | 4 | 1198.1 | airsacculitis |
|  | 6 | 1795.2 | airsacculitis |
| B50 | 2 | 133.8 | airsacculitis, litter in crop/gizzard, pericarditis |
|  | 2 | 151.8 | airsacculitis, dehydration, necrotic enteritis, pericarditis, perihepatitis |
|  | 2 | 216.7 | ascites, pericarditis |
| B10-2 | 2 | 144.3 | airsacculitis, dehydration, necrotic enteritis, pericarditis |
|  | 3 | 421.5 | airsacculitis, dehydration, necrotic enteritis, pericarditis, perihepatitis |
|  | 3 | 585.1 | airsacculitis |
| B10-5 | 1 | 44.7 | omphalitis |
|  | 1 | 55.6 | dehydration, omphalitis, pericarditis, perihepatitis |
|  | 2 | 186.8 | airsacculitis, pericarditis |
|  | 6 | 2073.2 | airsacculitis |
| B10-10 | 1 | 50.9 | airsacculitis, dehydration, gizzard erosion, litter in crop/gizzard, omphalitis |
|  | 1 | 70.7 | airsacculitis, dehydration, nonspecific enteritis, pericarditis |
|  | 2 | 170.7 | airsacculitis, dehydration, pericarditis, perihepatitis |
|  | 2 | 208.4 | airsacculitis, dehydration, litter in crop/gizzard, pericarditis, perihepatitis |
|  | 2 | 212.4 | airsacculitis, dehydration, litter in crop/gizzard, pericarditis |
|  | 2 | 231.7 | ascites, airsacculitis, joint enlargement, necrotic enteritis |
|  | 4 | 858.7 | gizzard erosion, litter in crop/gizzard, nonspecific enteritis |
|  | 6 | 2488.4 | airsacculitis |
