# Supplementary material for: Nutrient composition and safety evaluation of simulated isobutanol distillers dried grains with solubles and associated fermentation metabolites when fed to male Ross 708 broiler chickens (Gallus domesticus)
Source: PLoS One. 2019 Jul 8;14(7):e0219016. doi: 10.1371/journal.pone.0219016 (PMC6613701; doi:10.1371/journal.pone.0219016)
Supplement: S9 Table — (DOCX) [file pone.0219016.s009.docx]

S9 Table. Incidence and severity of histologic^1^ observations in the kidneys.

|  | eDDGS | B10 | B50 | B10-2 | B10-5 | B10-10 |
| --- | --- | --- | --- | --- | --- | --- |
| Number Examined | 25 | 25 | 25 | 25 | 25 | 25 |
| Basophilic Tubules | 25 | 25 | 25 | 25 | 25 | 25 |
| - minimal | 0 | 1 | 0 | 3 | 2 | 0 |
| - mild | 24 | 22 | 25 | 20 | 22 | 23 |
| - moderate | 1 | 2 | 0 | 2 | 1 | 2 |
| Infiltrate; Heterophilic | 1 | 0 | 1 | 1 | 0 | 0 |
| - minimal | 1 | 0 | 0 | 1 | 0 | 0 |
| - mild | 0 | 0 | 1 | 0 | 0 | 0 |
| Infiltrate; Mononuclear cell | 25 | 25 | 25 | 25 | 25 | 25 |
| - minimal | 6 | 7 | 7 | 5 | 7 | 3 |
| - mild | 19 | 17 | 16 | 19 | 17 | 20 |
| - moderate | 0 | 1 | 2 | 1 | 1 | 2 |
| Mineralization; Tubules^2^ | 6 | 5 | 7 | 4 | 3 | 5 |
| Vacuolization; Focal, Perivascular^3^ | 1 | 1 | 1 | 1 | 0 | 0 |

^1^Minimal grades were used for tissues within which the observation only slightly altered the normal and expected appearance of the organ/tissue. Mild grades were used for less than 25% involvement of the parenchyma. A moderate grade was used for conditions that were of were of sufficient severity or extent to include up to 50% of the parenchyma. Table reports observations where combined incidence across treatment groups was greater than 1. ^2^Histologic grade for all observations was minimal.
