# Supplementary material for: Nutrient composition and safety evaluation of simulated isobutanol distillers dried grains with solubles and associated fermentation metabolites when fed to male Ross 708 broiler chickens (Gallus domesticus)
Source: PLoS One. 2019 Jul 8;14(7):e0219016. doi: 10.1371/journal.pone.0219016 (PMC6613701; doi:10.1371/journal.pone.0219016)
Supplement: S10 Table — (DOCX) [file pone.0219016.s010.docx]

S10 Table. Incidence and severity of histologic^1^ observations in the skeletal muscle.

|  | eDDGS | B10 | B50 | B10-2 | B10-5 | B10-10 |
| --- | --- | --- | --- | --- | --- | --- |
| Number Examined | 25 | 25 | 25 | 25 | 25 | 25 |
| No Visible Lesions | 0 | 1 | 2 | 3 | 2 | 3 |
| Degeneration; Myofiber | 24 | 23 | 21 | 20 | 20 | 22 |
| - minimal | 8 | 4 | 11 | 5 | 4 | 6 |
| - mild | 10 | 13 | 9 | 9 | 5 | 9 |
| - moderate | 6 | 6 | 1 | 6 | 11 | 7 |
| Inflammation; Mixed | 18 | 22 | 18 | 20 | 20 | 17 |
| - minimal | 13 | 17 | 16 | 13 | 14 | 11 |
| - mild | 5 | 5 | 6 | 4 | 5 | 6 |
| - moderate | 0 | 0 | 0 | 3 | 1 | 0 |
| Regeneration; Myofiber | 7 | 4 | 4 | 10 | 8 | 4 |
| - minimal | 6 | 2 | 3 | 6 | 4 | 3 |
| - mild | 1 | 2 | 1 | 2 | 3 | 1 |
| - moderate | 0 | 0 | 0 | 2 | 1 | 0 |

^1^Minimal grades were used for tissues within which the observation only slightly altered the normal and expected appearance of the organ/tissue. Mild grades were used for less than 25% involvement of the parenchyma. A moderate grade was used for conditions that were of were of sufficient severity or extent to include up to 50% of the parenchyma. Table reports observations where combined incidence across treatment
