# Supplementary material for: Nutrient composition and safety evaluation of simulated isobutanol distillers dried grains with solubles and associated fermentation metabolites when fed to male Ross 708 broiler chickens (Gallus domesticus)
Source: PLoS One. 2019 Jul 8;14(7):e0219016. doi: 10.1371/journal.pone.0219016 (PMC6613701; doi:10.1371/journal.pone.0219016)
Supplement: S11 Table — (DOCX) [file pone.0219016.s011.docx]

S11 Table. Incidence and severity of histologic^1^ observations in the bursa of Fabricius.

|  | eDDGS | B10 | B50 | B10-2 | B10-5 | B10-10 |
| --- | --- | --- | --- | --- | --- | --- |
| Number Examined | 24^2^ | 25 | 25 | 25 | 25 | 25 |
| No Visible Lesions | 1 | 2 | 6 | 2 | 2 | 10 |
| Cyst | 2 | 3 | 4 | 4 | 2 | 4 |
| - minimal | 1 | 1 | 1 | 1 | 1 | 1 |
| - mild | 1 | 1 | 2 | 3 | 1 | 2 |
| - moderate | 0 | 1 | 1 | 0 | 0 | 1 |
| Edema^3^ | 1 | 0 | 1 | 0 | 0 | 0 |
| Increased fibrous tissue | 6 | 0 | 4 | 4 | 2 | 0 |
| - minimal | 5 | 0 | 3 | 4 | 1 | 0 |
| - mild | 1 | 0 | 0 | 0 | 1 | 0 |
| - moderate | 0 | 0 | 1 | 0 | 0 | 0 |
| Infiltration; Heterophilic^3^ | 1 | 2 | 0 | 0 | 1 | 2 |
| Inflammation; Subacute/chronic, Focal | 3 | 1 | 2 | 3 | 3 | 4 |
| - minimal | 1 | 0 | 2 | 2 | 3 | 3 |
| - mild | 2 | 1 | 0 | 1 | 0 | 1 |
| Necrosis; Lymphoid | 22 | 23 | 17 | 21 | 23 | 12 |
| - minimal | 15 | 13 | 13 | 17 | 18 | 19 |
| - mild | 7 | 10 | 4 | 4 | 5 | 3 |

^1^Minimal grades were used for tissues within which the observation only slightly altered the normal and expected appearance of the organ/tissue. Mild grades were used for less than 25% involvement of the parenchyma. A moderate grade was used for conditions that were of were of sufficient severity or extent to include up to 50% of the parenchyma. Table reports observations where combined incidence across treatment groups was greater than 1. ^2^Bursa of Fabricius from 1 broiler from the eDDGS treatment was missing. ^3^Histologic grade for all observations was minimal.
