# Supplementary material for: Nutrient composition and safety evaluation of simulated isobutanol distillers dried grains with solubles and associated fermentation metabolites when fed to male Ross 708 broiler chickens (Gallus domesticus)
Source: PLoS One. 2019 Jul 8;14(7):e0219016. doi: 10.1371/journal.pone.0219016 (PMC6613701; doi:10.1371/journal.pone.0219016)
Supplement: S12 Table — (DOCX) [file pone.0219016.s012.docx]

S12 Table. Incidence and severity of histologic^1^ observations in the lungs.

|  | eDDGS | B10 | B50 | B10-2 | B10-5 | B10-10 |
| --- | --- | --- | --- | --- | --- | --- |
| Number Examined | 25 | 25 | 25 | 25 | 25 | 25 |
| No Visible Lesions | 5 | 9 | 7 | 9 | 5 | 12 |
| Cellular debris; Lumen, Parabronchus^2^ | 1 | 0 | 2 | 0 | 0 | 0 |
| Hyperplasia; Lymphoid, Peribronchial | 19 | 16 | 17 | 15 | 19 | 13 |
| - minimal | 14 | 12 | 14 | 11 | 13 | 13 |
| - mild | 4 | 4 | 3 | 4 | 6 | 0 |
| - moderate | 1 | 0 | 0 | 0 | 0 | 0 |
| Inflammation; Subacute/chronic; Focal | 5 | 3 | 5 | 4 | 9 | 3 |
| - minimal | 3 | 2 | 3 | 2 | 4 | 2 |
| - mild | 2 | 0 | 2 | 2 | 3 | 1 |
| - moderate | 0 | 1 | 0 | 0 | 2 | 0 |

^1^ Minimal grades were used for tissues within which the observation only slightly altered the normal and expected appearance of the organ/tissue. Mild grades were used for less than 25% involvement of the parenchyma. A moderate grade was used for conditions that were of were of sufficient severity or extent to include up to 50% of the parenchyma. Table reports observations where combined incidence across treatment groups was greater than 1. ^2^Histologic grade for all observations was minimal.
